# Depression in the Elderly. Consensus Statement of the Spanish Psychogeriatric Association

**DOI:** 10.3389/fpsyt.2020.00380

**Published:** 2020-05-20

**Authors:** Luis Agüera-Ortiz, María Dolores Claver-Martín, María Dolores Franco-Fernández, Jorge López-Álvarez, Manuel Martín-Carrasco, María Isabel Ramos-García, Manuel Sánchez-Pérez

**Affiliations:** ^1^Servicio de Psiquiatría, Instituto de Investigación i+12, Hospital Universitario 12 de Octubre, Madrid, Spain; ^2^Centro de Investigación Biomédica en Red de Salud Mental (CIBERSAM), Madrid, Spain; ^3^Madrid Salud, Ayuntamiento de Madrid, Madrid, Spain; ^4^Psychiatry Department, University of Sevilla, Seville, Spain; ^5^FIDMAG Research Foundation (CIBERSAM), Barcelona, Spain; ^6^Instituto de Psiquiatría y Salud Mental, Hospital Clínico San Carlos, Instituto de Investigación Sanitaria del Hospital Clínico San Carlos (IdISSC), Madrid, Spain; ^7^Unidad de Psiquiatría Geriátrica, Hospital Sagrat Cor. Martorell, Barcelona, Spain

**Keywords:** depression, elderly, consensus statement, clinical recommendations, antidepressant drugs, treatment-resistance, comorbidity

## Abstract

**Introduction:**

Present knowledge about depression in the elderly is still scarce and often controversial, despite its high frequency and impact. This article reports the results and most relevant conclusions of a Delphi-based consensus on geriatric depression promoted by the Spanish Psychogeriatric Association.

**Methods:**

A 78-item questionnaire was developed by 7 highly specialized geriatric psychiatrists and was evaluated using the Modified Delphi technique in two rounds answered by 35 psychiatrists with an extensive expertise in geriatric depression. The topics and number of questions (in brackets) covered were: concepts, clinical aspects, and risk factors (12); screening and diagnosis (7); psychotic depression (17); depression and dementia (5); antidepressant drug treatment (18); non-pharmacological biological treatments (5); psychotherapeutic treatments (4); comorbidity and preventive aspects (6); professional training needed (4). In addition, the expert panel’s opinion on the antidepressants of choice in 21 common comorbid conditions and on different strategies to approach treatment-resistant cases in terms of both efficacy and safety was assessed.

**Results:**

After the two rounds of the Delphi process, consensus was reached for 59 (75.6%) of the 78 items. Detailed recommendations are included in the text. Considering pharmacological treatments, agomelatine was the most widely mentioned drug to be recommended in terms of safety in comorbid conditions. Desvenlafaxine, sertraline, and vortioxetine, were the most frequently recommended antidepressants in comorbid conditions in general. Combining parameters of efficacy and safety, experts recommended the following steps to address cases of treatment resistance: 1. Escalation to the maximum tolerated dose; 2. Change of antidepressant; 3. Combination with another antidepressant; 4. Potentiation with an antipsychotic or with lamotrigine; 5. Potentiation with lithium; 6. Potentiation with dopamine agonists or methylphenidate

**Discussion and Conclusions:**

Consensus was reached for a high number of items as well as for the management of depression in the context of comorbid conditions and in resistant cases. In the current absence of sufficient evidence-based information, our results can be used to inform medical doctors about clinical recommendations that might reduce uncertainty in the diagnosis and treatment of elderly patients with depressive disorders.

## Introduction

Depression in the elderly can be considered a real public health problem, because of its frequency, its clinical implications, and the great suffering that it causes (1, 2). To date, unfortunately, the degree of knowledge by the health professionals involved in its diagnosis and treatment is far from what would be desirable.

The irrefutable epidemiological data pertaining to the increase in the proportion of elderly population and the high prevalence of depression in such age group have led to a growing demand for care that requires efficient, evidence-based responses (3–5).

However, until recent decades, neither primary care physicians nor specialists have given this disease due consideration, and this is reflected in the scarce literature on the topic. While professionals demand information, what can be found in the specialized literature, be it articles, books or clinical practice guidelines, does not frequently meet their expectations.

Significant advances have been made regarding the neurobiology of depression in older people, from many points of view, from basic science (6) to the influence of psychological issues (7). The link with neurodegenerative diseases has also been extensively explored (8, 9). Nevertheless, research performed in the area is clearly insufficient. Furthermore, older adults are often excluded from pharmacotherapeutic clinical trials. Because of all this, physicians’ decision making is permanently affected by the lack of sound scientific bases and by the use of information drawn from non-geriatric population groups. This results in frequent disagreement regarding the divergent findings of the available research, which partly explains the variability of professional judgment and clinical habits among specialists in the area.

One way to remedy this situation is to draw up clinical practice guides and build expert consensuses. In recent years, the number of guides addressing geriatric depression therapy has slightly increased. However, the scarcity of documents that approach depression in the elderly from a broader perspective that goes beyond treatment is still remarkable (10–16).

The Spanish Psychogeriatric Association drew up a first consensus document on geriatric depression that was presented in 2011 (17). However, it is now deemed necessary to update its proposals and contents. This second edition is not a revised version of the first one, but constitutes a new work practically from scratch.

The common goal of both projects was and is to reach consensus on a proposal for professional criteria and clinical recommendations to reduce uncertainty in the diagnosis and treatment of elderly patients with depressive disorders. As with the first edition of the Consensus, the latest version of the Delphi method, an agreement of expert’s technique that is reliable and has a long tradition of use in biomedicine has been used.

The main purpose of this project was to explore the opinion of a panel of experts in psychogeriatrics and reach sufficient professional consensus based on a battery of criteria and recommendations regarding depression management in elderly patients in the light of the available scientific knowledge and the panel’s clinical expertise.

The study consists of three parts: an actual consensus on different issues regarding the management of depression in the elderly, using the Delphi method; and two specific analyses that follow two different approaches, one addressing the choice of the most adequate antidepressant drug according to existing comorbidities and another on strategies for cases of inadequate response to antidepressant therapy.

This article reports the results and most relevant conclusions of the study, hoping that they might be of practical interest not only for specialists in psychiatry, but also for other professionals that care for the elderly, such as geriatricians, primary care physicians, physicians working at nursing homes, neurologists or psychologists, beyond the territorial context where it was carried out.

## Materials and Methods

### Delphi Study

The first part of the consensus document is a study using the Delphi method. Such technique is based on the answers to a questionnaire previously drawn up by a scientific committee of experts or “core” provided to a group of experts in the same area, known as panellists, that is assessed more than once. The purpose of the series of questionnaires is to reduce variability in answers as far as possible.

The expert committee for this consensus consisted of seven leading members of the Spanish Psychogeriatric Association, and the panel of experts was made up of 35 psychiatrists with clinical expertise and specific professional recognition in the approach of depression in elderly patients, selected in accordance with the criteria of the expert committee following a *snowball* sampling procedure (18). The various geographical areas of Spain were represented thus assuring a lack of regional bias. Some of the experts, but not all, had taken part in the first edition.

The scientific committee drew up a series of items for the survey, designed in the form of statements (positive and negative). A first list of these statements was built using in part those of the first edition of the consensus and many other new ones. In some instances, questions that achieved a good degree of agreement in the first edition were not repeated in the second in order to keep a manageable total number that could be adequately answered by the experts. The items included in the final questionnaire were the product of a process of consensus achieved through email exchanges among the members and a face-to-face meeting.

The final version of the survey contains 78 items structured into nine topic areas. The items by topic area were:

**Table:** 

Topic area	Number of items
1.- Concepts, clinical aspects, and risk factors	12
2.- Screening and diagnosis	7
3.- Psychotic depression	17
4.- Depression and dementia	5
5.- Antidepressant drug treatment	18
6.- Non-pharmacological biological treatments (fundamentally ECT)	5
7.- Psychotherapeutic treatments	4
8.- Comorbidity and preventive aspects	6
9.- Professional training to address depression in the elderly	4
TOTAL	78

The two rounds of the Delphi process were performed in the first trimester of 2018. Once the Delphi method had been applied, results were organized and discussed by the core of experts, checking them against the existing scientific literature.

All the results and conclusions have been published in an extensive monograph that is available on the website of the Spanish Psychogeriatric Association (www.sepg.es). This article presents the most relevant aspects of such study.

The Delphi method is a structured technique for a process of group communication by members who are generally geographically scattered, and is effective in allowing a group of individuals, as a whole, to address a complex problem. The forecasting capacity of the Delphi method is based on the systematic use of an intuitive opinion expressed by a group of experts (19, 20).

The modified Delphi method (21) that has been used in this study was applied in two successive rounds of a closed-ended questionnaire constructed by the core of experts. In between rounds, each participant was informed of the processing of the intermediate results achieved and the comments made by the panel, which offers individuals the opportunity to contrast their opinions with those of the other panelists and reconsider their position. The main characteristics of a Delphi study are anonymity, possible panelist heterogeneity, iteration, and controlled feedback of group answers indicating the level of agreement or disagreement reached, as well as the statistical validation of the final result. For interaction between panelists and the scientific committee, this study used a specifically designed website where participants entered their answers and received the corresponding feedback on them.

Statements (items) were assessed using a single ordinal nine-point Likert type scale, according to the UCLA-Rand Corporation format that is currently used for healthcare technology assessment (19, 21, 22). Response categories are defined by linguistic descriptors of agreement/disagreement with the statements presented, as follows:

- 1-3: I disagree with the statement (the lower the score, the greater the level of disagreement).
- 4-6: I neither agree nor disagree with the statement; I do not have a well-defined criterion on the issue (choose 4 or 6 if closer to disagreement or to agreement, respectively).
- 7-9: I agree with the statement (the higher the score, the greater the level of agreement).

**Figure 1:**
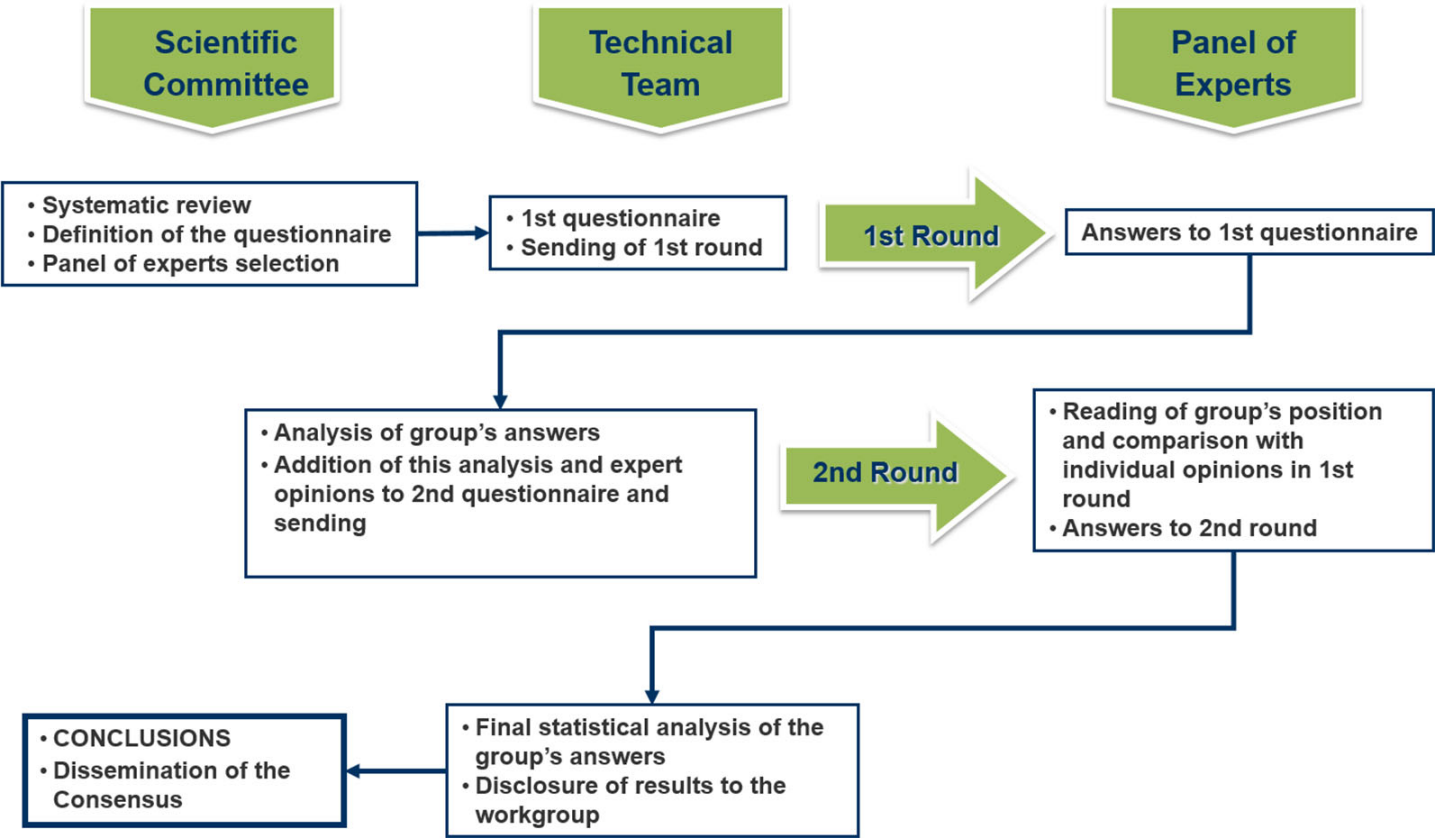
Phases of the Delphi study.

The phases of the Delphi study are shown in **Figure 1**.

#### Statistical Analysis of the Delphi Study Results

For each item of the questionnaire, the mean of the panelists’ scores and the confidence interval for the average at a 95% confidence level were calculated.

Interpretation according to statistical estimators was performed using the following explicit criteria for achieved or failed consensus:

- The higher the average (closer to 9) for each item, the stronger the group’s agreement with the statement expressed by the item.
- The lower the average (closer to 1) for each item, the stronger the group’s disagreement with the statement expressed by the item.
- The lower the confidence interval, the greater the unanimity in the group’s agreement or disagreement opinions.
- If the confidence interval encompasses the value 5 (“neither agree nor disagree”), the group does not reach unanimous consensus in either direction. In such case, a description of the causes for such lack of consensus in any direction of the side values will be described (there are either markedly different opinions among panelists, or most of the group claims to have no definitive criterion on the item).

The analysis of group opinion and the type of consensus reached on each of the questions posed was conducted using the position of the median of group scores (score zone: [1-3] [4-6] [7-9]) and the “level of agreement” reached by the respondents, according to the following criteria:

Item consensus was established when the panel showed “**concordance”** of opinion, that is, when experts scoring outside the three-point zone ([1-3], [4-6], [7-9]) that includes the median accounted for less than one third of the respondents. In such case, the median value defined the type of group consensus reached: majority “**disagreement**” with the item, if the median was ≤ 3, or majority “**agreement**” with the item if the median was ≥ 7. When the median was within the 4-6 zone, items were considered “**unclear**” for a representative majority of the group.

Conversely, when the scores of one third or more of the panelists fell within the [1-3] zone and those of another third within the [7-9] zone, “discrepancy” in criterion was established. The remaining items that obtained neither consistency nor discrepancy were considered of “indeterminate” level of consensus.

All the items on which the group did not reach clear consensus in favor or against the issue (unclear items, those where disagreement can be observed and those where consensus was indeterminate) were proposed for reconsideration by the panelists in the second round of the Delphi. Items on which there was high dispersion of opinions among the respondents, with interquartile ratings ≥ 4 points (from p25 to p75) were also subjected to reassessment.

In between both rounds, panelists were informed of the detailed distribution of the group’s answers to the first survey (using bar graphs), and they were provided with the comments and explanations contributed by each participant. After reviewing such information, they were asked for a new personal assessment of the items on which no consensus had been reached in the first round.

The findings are presented using a color code that accounts for the final positioning of group opinion on each of the assessed items (a clinical criterion or recommendation):

Representation of findings is done as follows:

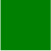
 **CONSENSUS AGREEMENT WITH ITEMS** (The group ACCEPTS the item by consensus). Marked in green color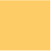 **NO CONSENSUS ON ITEMS AFTER THE TWO INTERACTION ROUNDS** (The group does not have a sufficiently homogeneous opinion). Marked in orange color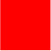 **CONSENSUS DISAGREEMENT WITH ITEMS** (The group REJECTS the item by consensus). Marked in red color

### Choice of Antidepressant Drug According to Existing Medical Comorbidities

Preferences regarding the use of different antidepressants in patients with various medical comorbidities that are frequent in older adults were subjected to the consensus process and collected during the first round of the Delphi study. All the modern antidepressants available in Spain at the time of the fieldwork were included. Regarding the oldest drugs, monoamine oxidase inhibitors were not included, and of the tricyclics, only nortriptyline -which is possibly the most widely supported tricyclic choice for use in the elderly population- was assessed.

The observations in this section mostly refer to the safety of antidepressants in the presence of each of the listed situations and not to the efficacy of the drug under such circumstances, except in the generic case of chronic pain patients.

The comorbidity situations proposed were as follows:

1. Ischemic cardiomyopathy/infarction
2. Arrhythmias
3. Hypertension
4. Anticoagulated patient
5. Diabetes
6. Dyslipidemia
7. Obesity
8. Appetite and weight loss
9. Significant constipation
10. Risk of gastrointestinal bleeding
11. Risk of hyponatremia
12. Drowsiness
13. Falls
14. Alcohol abuse
15. Sexual dysfunction
16. Glaucoma
17. Risk of epileptic seizures
18. Stroke
19. Parkinson’s disease and extrapyramidal disorders
20. Cognitive impairment, dementia, Alzheimer’s disease
21. Pain disorders

For each of these situations, the expert was asked to choose among the following options:

- Drug *contraindicated* in such clinical situation
- Drug *to be avoided as far as possible*: it has some disadvantages compared to other options
- Drug that *could be used*: it has no particular advantages or disadvantages
- Drug that is a *good alternative*: it has certain advantages over the rest of options
- Drug *of choice* in this clinical situation
- No opinion about it

To facilitate interpretation, answers have been arranged according to the following criteria for each of the given comorbidity situations:

**Table:** 

**Specifically recommended antidepressants**	Selected as drug of choice by ≥ 6 panelists Selected as contraindicated drug by < 6 panelists
**Antidepressants that are a reasonable option**	Selected by ≥ 6 panelists Selected as contraindicated drug by < 6 panelists
**Antidepressants to be avoided**	Selected as contraindicated drug by ≥ 6 panelists

### Strategies for Treatment-Resistant Depression or Inadequate Response to Antidepressant Therapy

The last part of the consensus involved assessing the different strategies to approach cases where depression was resistant or where adequate response to the originally prescribed antidepressant treatment was not achieved, in terms of efficacy and of safety separately. This information was collected during the first round of the Delphi study.

Experts were provided with a list of 9 possible strategies to rank in order of preference. We opted for a fairly open definition of inadequate response or resistance to treatment. Experts were asked to order the 9 options in the form of progressive steps in case the previous one failed, in an attempt to reflect routine clinical practice.

Such 9 strategies were as follows:

Escalation to maximum dosage
Change to an antidepressant from a different group
Change to an antidepressant of the same group
Combination with another antidepressant
Potentiation with lithium
Potentiation with an antipsychotic drug
Potentiation with lamotrigine
Potentiation with a dopamine agonist
Potentiation with methylphenidate
Each of these options was to be assessed on a 1 to 9 scale, where 1 = first to use; and 9 = last to use. Besides, they were asked to rank the different options in order of safety on another 1 to 9 scale, where 1 = safest; and 9 = least safe. Results were analyzed by summarizing the number of votes.

## Results

### Delphi Consensus

After the two rounds of the Delphi process, consensus was reached for 59 (75.6%) of the 78 items that made up the survey. Such percentage comprises the 47 (60.2%) items where there was consensus in accepting the statement proposed and the 12 (15.4%) items where there was consensus in rejecting it. No consensus was reached on 19 items (24.4%).

The number and percentage of items (in brackets) for which a consensus was reached in each section were as follows:

Concepts, clinical aspects, and risk factors: Consensus in 11 (92%) out of 12 items. Screening and diagnosis: Consensus in 6 (85,7%) out of 7 items. Psychotic depression: Consensus in 10 (59%) out of 17 items. Depression and dementia: Consensus in all 5 (100%) items. Antidepressant drug treatment: Consensus in 11 (61%) out of 18 items. Non-pharmacological biological treatments (fundamentally ECT): Consensus in 4 (80%) out of 5 items. Psychotherapeutic therapies: Consensus in 2 (50%) out of 4 items. Comorbidity and preventive aspects Consensus in all 6 (100%) items. Professional training to address depression in the elderly: Consensus in all 4 (100%) items.

**Table 1** provides a summary of the results of the different question groups, distinguishing between items for which consensus was reached and those for which it was not. Among the former, there is also a distinction in the central columns between items with agreement consensus and items where there is consensus in rejecting the statement.

**Table 1:** Degree of consensus for the items grouped into sections.

Section	Total items	Items that reached consensus (%)			Items that did not reach consensus (%)
			Items with agreement consensus (%)	Items with disagreement` consensus (%)	
Concepts, clinical aspects and risk factors	12	**11** (**92%**)			**1** (**8%**)
			11 (92%)	0 (0%)	
Screening and diagnosis	7	**6** (**85.7%**)			**1** (**14,3%**)
			5 (71,4%)	1 (14,3%)	
Psychotic depression	17	**10** (**59%**)			**7** (**41%**)
			6 (35%)	4 (24%)	
Depression and dementia	5	**5** (**100%**)			**0** (**0%**)
			4 (80%)	1 (20%)	
Antidepressant drug treatment	18	**11** (**61%**)			**7** (**39%**)
			9 (50%)	2 (11%)	
Non-pharmacological biological treatment (fundamentally ECT)	5	**4** (**80%**)			**1** (**20%**)
			2 (40%)	2 (40%)	
Psychotherapeutic therapies	4	**2** (**50%**)			**2** (**50%**)
			2 (50%)	0 (0%)	
Comorbidity and preventive aspects	6	**6** (**100%**)			**0** (**0%**)
			5 (83%)	1 (17%)	
Professional training to address depression in the elderly	4	**4** (**100%**)			**0** (**0%**)
			3 (75%)	1 (25%)	
Total	78	**59 (75.6%)**			**19 (24.4%)**
			47 (60.2%)	12 (15.4%)	

**Figure 2:**
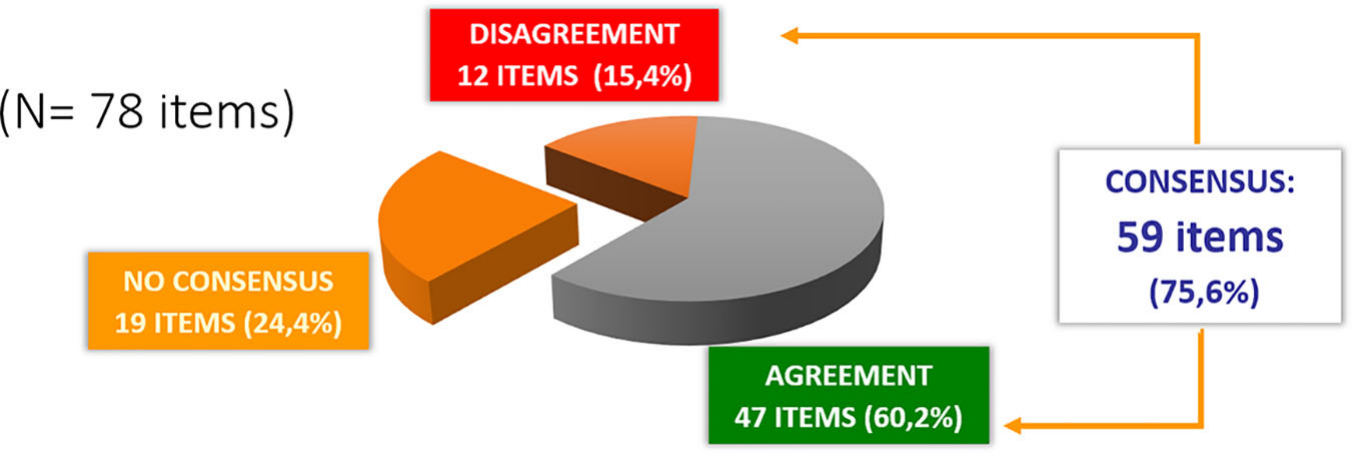
Global results: Degree of consensus.

**Figure 2** shows a summary of the overall level of consensus achieved.

The degree of consensus on the different items grouped into nine topic areas is shown in **Tables 2**–**10**. Arithmetical means and confidence intervals for each item appear in the **Supplementary Material**.

**Table 2:** Topic area 1—Concepts, clinical aspects, and risk factors.

	Consensus Disagreement	No consensus	Consensus Agreement
1.1. Late-onset depression (first episode in old age rather than in adulthood) is a clinically useful concept in psychiatry.			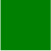
1.2. Vascular depression is a clinically useful concept in psychiatry.			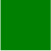
1.3. Depressive pseudodementia is a clinically useful concept in psychiatry.		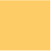	
1.4. Depression in the elderly, as compared to adults, is more specifically associated with higher levels of anxiety.			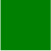
1.5. Depression in the elderly, as compared to adults, is more specifically associated with higher levels of hypochondriac symptomatology.			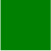
1.6. Depression in the elderly, as compared to adults, is more specifically associated with less likelihood to express sadness.			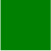
1.7. Depression in the elderly, as compared to adults, is more specifically associated with higher levels of suicidal ideation.			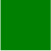
1.8. Depression in the elderly, as compared to adults, is more specifically associated with greater impact on daily life.			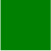
1.9. Frailty states in the transition from autonomy to dependence entail a significant risk factor for depression in old age.			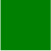
1.10. Disclosure of a dementia diagnosis is a significant risk factor for depression in old age.			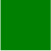
1.11. Severe physical diseases in older adults are a higher risk factor for suicide in men than in women.			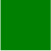
1.12. Non-depressed older adults with thoughts of death require clinical care.			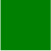

**Table 3:** Topic area 2—Screening and diagnosis.

	Consensus disagreement	No consensus	Consensus agreement
2.1. The diagnostic criteria of standard nosologies (ICD 11, DSM5) are adequate to diagnose and classify depressive disorders in the elderly.	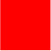		
2.2. Specific diagnostic criteria for depressive disorders in the elderly population are necessary.			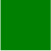
2.3. Teleassistance services should include some type of screening system for depression.			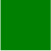
2.4. ALL elderly patients living in nursing homes should be systematically screened for depression.			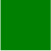
2.5. ALL elderly patients should be systematically screened for depression in Primary Care.			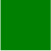
2.6. Any diagnostic process in late-onset depression should include laboratory tests.			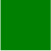
2.7. Any diagnostic process in late-onset depression should include neuroimaging testing.		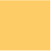	

**Table 4:** Topic area 3—Psychotic depression.

	Consensus disagreement	No consensus	Consensus agreement
3.1. Negativistic/oppositional behaviors in depressed older adults point towards the presence of psychotic depression.		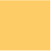	
3.2. The onset of psychotic symptoms in depressed older adults involves a higher risk for evolving into dementia.			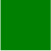
3.3. The onset of psychotic symptoms in depressed older adults involves a greater risk for suicide.			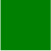
3.4. ECT is the first therapeutic option to treat psychotic depression in the elderly.		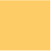	
3.5. Combined treatment with antidepressants and antipsychotics is the first therapeutic option to treat psychotic depression in the elderly.			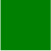
3.6. ECT should ONLY be used to treat psychotic depression in older adults when there is lack of response to pharmacological treatment.	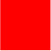		
3.7. In psychotic depression in the elderly, dual-action antidepressants are preferable to SSRIs.			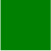
3.8. When pharmacological treatment fails, ECT is the best option to treat psychotic depression in the elderly.			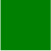
3.9. In non-life-threatening psychotic depression in the elderly that does not respond to pharmacological treatment, ECT should be prescribed no later than 6 weeks.		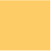	
3.10. In non-life-threatening psychotic depression in the elderly that does not respond to pharmacological treatment, ECT should be prescribed no later than 12 weeks.		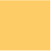	
3.11. In non-life-threatening psychotic depression in the elderly that does not respond to pharmacological treatment, ECT should be prescribed no later than 24 weeks.	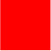		
3.12. After good response to ECT in the acute phase, pharmacological treatment combined with continuation/maintenance ECT is the treatment of choice to prevent early relapse and recurrence.			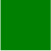
3.13. After good response to ECT in the acute phase, pharmacological-only continuation/maintenance treatment is the treatment of choice to prevent early relapse and recurrence.		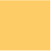	
3.14. After good response to ECT in the acute phase, continuation/maintenance ECT therapy-only is the treatment of choice to prevent early relapse and recurrence.		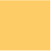	
3.15. After good response to ECT in the acute phase, the addition of lithium to the combined antidepressant-antipsychotic drug therapy is the treatment of choice to prevent early relapse and recurrence.	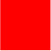		
3.16. During continuation/maintenance treatment of psychotic depression, the antipsychotic should be maintained as long as the antidepressant.	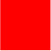		
3.17. In psychotic depression in the elderly, pharmacological treatment should be maintained indefinitely even if there has only been one episode.		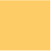	

**Table 5:** Topic area 4—Depression and dementia.

	Consensus disagreement	No consensus	Consensus agreement
4.1. The criteria to diagnose depression in dementia/major neurocognitive disorder are well defined and clinically useful.	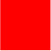		
4.2. It is necessary to establish differentiated depression criteria for the different diseases or clinical conditions that may involve dementia/major neurocognitive disorder (for example, Alzheimer’s disease, Parkinson’s disease, frontotemporal dementia, etc.).			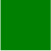
4.3. It is necessary to establish differentiated depression criteria for the different phases of dementia/major neurocognitive disorder.			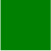
4.4. Antidepressant drugs are effective in the treatment of depression in dementia.			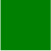
4.5. Psychological therapies are effective in the treatment of depression in dementia.			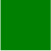

**Table 6:** Topic area 5—Antidepressant drug treatment.

	Consensus disagreement	No consensus	Consensus agreement
5.1. Subclinical depression requires pharmacological treatment.		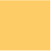	
5.2. When prescribing antidepressants for elderly patients, laboratory tests should be carried out at the beginning of treatment.			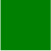
5.3. When prescribing an antidepressant for an elderly patient, an EKG should be performed at the beginning of treatment.			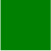
5.4. In general, SSRIs antidepressants are first choice treatment drugs for depression in the elderly.			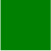
5.5. In general, dual-action antidepressants are first choice drugs for depression in the elderly.		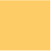	
5.6. Dual-action antidepressants achieve higher levels of effectiveness in the treatment of depression in the elderly as compared to SSRIs.			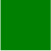
5.7. Dietary supplements (Omega 3, DHA…) are effective in improving depression in the elderly.		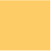	
5.8. Antidepressants have a slower onset of action in the elderly than in younger adults.			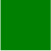
5.9. The introduction of antidepressants increases the risk for suicide in depressed elderly patients at the beginning of treatment.		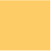	
5.10. The elderly tolerate dual-action antidepressants better than SSRIs.		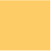	
5.11. Sexual dysfunction caused by antidepressants is a problem for elderly patients.			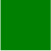
5.12. After a depressive episode in an elderly patient, treatment at effective doses should be maintained for 6 months.		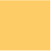	
5.13. After a depressive episode in an older adult, treatment at effective doses should be maintained for 1 year.			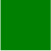
5.14. After a depressive episode in an older adult, treatment at effective doses should be maintained for 2 years.		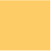	
5.15. After a depressive episode in an older adult, treatment at effective doses should be maintained for 3 to 4 years.	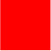		
5.16. In depression in elderly patients, indefinite pharmacological therapy should be stablished after the first episode.	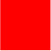		
5.17. In depression in elderly patients, indefinite pharmacological therapy should be stablished after the second episode.			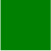
5.18. In depression in elderly patients, indefinite pharmacological therapy should be stablished after the third episode.			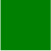

**Table 7:** Topic area 6—Non-pharmacological biological treatments (fundamentally ECT).

	Consensus disagreement	No consensus	Consensus agreement
6.1. ECT is indicated for vascular depression.			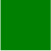
6.2. ECT is indicated for depression in elderly patients with dementia.			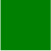
6.3. Bilateral ECT should only be used when unilateral ECT yields unsatisfactory results.	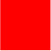		
6.4. Cognitive effects associated with ECT significantly limit its indication for depression in elderly patients.	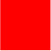		
6.5. Transcranial magnetic stimulation should be regarded as a therapeutic option for resistant depression in elderly patients.		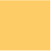	

**Table 8:** Topic area 7—Psychotherapeutic treatments.

	Consensus disagreement	No consensus	Consensus agreement
7.1. The efficacy of psychotherapy in geriatric depression is at least equal to that of pharmacological treatments.		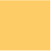	
7.2. Psychotherapy is less effective in the elderly than in adults.		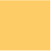	
7.3. In geriatric depression, the presence of cognitive impairment/mild dementia does not limit the use of psychotherapy.			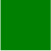
7.4. Psychotherapy is effective in the treatment of subclinical geriatric depression.			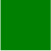

**Table 9:** Topic area 8—Comorbidity and preventive aspects.

	Consensus disagreement	No consensus	Consensus agreement
8.1. In cases of late-onset depression it is necessary to consider the subsequent development of a neurodegenerative disorder (dementia, Parkinson’s disease, etc.).			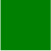
8.2. In cases of late-onset depression it is necessary to consider the subsequent development of a major medical condition (cancer, cardiopathies, etc.).			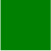
8.3. Elderly patients living in nursing homes have access to the same therapies (antidepressant treatment, psychotherapies, ECT, etc.) as those living in the community.	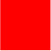		
8.4. Teleassistance services are useful to reduce the risk for suicide in the elderly.			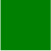
8.5. Physical exercise has a significant protective effect against depression in old age.			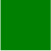
8.6. Interventions aimed at reducing social isolation are significant and effective strategies in the prevention of depression in the elderly.			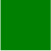

**Table 10:** Topic area 9—Professional training to address depression in the elderly.

	Consensus disagreement	No consensus	Consensus agreement
9.1. The training received by residents in psychiatry is currently insufficient to competently address depressive disorders in older adults.	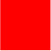		
9.2. Psychiatrists require specific training to competently address depressive disorders in older adults.			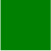
9.3. Detection of depression in older adults is currently noticeably below optimal and desirable levels.			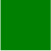
9.4. Because of its complexity, depression on older adults should be mainly treated by specialists in psychiatry.			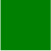

### Antidepressant Selection According to Medical Comorbidities

**Table 11** gathers all the recommendations regarding individual drugs for each of the clinical situations proposed.

**Table 11:** Individual drug recommendations for different comorbidity situations.

	Recommended	Reasonable option	To be avoided
Ischemic cardiomyopathy/infarction	Sertraline, agomelatine	Desvenlafaxine, venlafaxine, fluvoxamine, vortioxetine, mirtazapine	Citalopram/escitalopram, reboxetine, nortriptyline
Arrhythmias	Sertraline, agomelatine	Desvenlafaxine, fluoxetine, fluvoxamine, vortioxetine, mirtazapine	Citalopram/escitalopram, bupropion, reboxetine, nortriptyline
Hypertension	Sertraline, agomelatine	Citalopram/escitalopram, fluoxetine, fluvoxamine, paroxetine, vortioxetine, mirtazapine	Venlafaxine
Anticoagulated patient		Desvenlafaxine, venlafaxine, duloxetine, sertraline, citalopram/escitalopram, mirtazapine, bupropion, agomelatine, vortioxetine	
Diabetes		Desvenlafaxine, venlafaxine, duloxetine, fluoxetine, sertraline, citalopram/escitalopram, agomelatine, vortioxetine, bupropion, reboxetine	
Dyslipidemia		Fluoxetine, sertraline, citalopram/escitalopram, duloxetine, bupropion, vortioxetine, agomelatine	
Obesity	Fluoxetine, bupropion, agomelatine	Desvenlafaxine, venlafaxine, duloxetine, citalopram/escitalopram, fluvoxamine, sertraline, vortioxetine, reboxetine	Mirtazapine
Appetite and weight loss	Mirtazapine	Desvenlafaxine, venlafaxine, duloxetine, paroxetine, sertraline, citalopram/escitalopram, nortriptyline	Fluoxetine
Significant constipation		Fluoxetine, fluvoxamine, sertraline, citalopram/escitalopram, bupropion, agomelatine, vortioxetine	
Risk of gastrointestinal bleeding		Desvenlafaxine, venlafaxine, mirtazapine, bupropion, nortriptyline	Fluoxetine, citalopram/escitalopram
Risk of hyponatremia		Desvenlafaxine, venlafaxine, duloxetine, mirtazapine, bupropion, agomelatine, vortioxetine	Fluoxetine, citalopram/escitalopram
Drowsiness	Bupropion	Desvenlafaxine, venlafaxine, fluoxetine, sertraline, citalopram/escitalopram, reboxetine, agomelatine, vortioxetine	Mirtazapine
Falls		Desvenlafaxine, venlafaxine, duloxetine, fluoxetine, sertraline, citalopram/escitalopram, bupropion, vortioxetine	Nortriptyline
Alcohol abuse		Desvenlafaxine, venlafaxine, duloxetine, fluoxetine, paroxetine, Sertraline, citalopram/escitalopram, mirtazapine, bupropion, vortioxetine	
Sexual dysfunction	Agomelatine, bupropion, mirtazapine	Reboxetine, vortioxetine	Fluoxetine, paroxetine
Glaucoma		Desvenlafaxine, sertraline, citalopram/escitalopram, bupropion, agomelatine, vortioxetine, tianeptine	nortriptyline, venlafaxine
Risk of epileptic seizures		Mirtazapine	Nortriptyline, bupropion
Stroke		Desvenlafaxine, sertraline, citalopram/escitalopram, agomelatine, vortioxetine, mirtazapine	
Parkinson’s disease and extrapyramidal disorders	Bupropion	Desvenlafaxine, venlafaxine, duloxetine, sertraline, agomelatine, vortioxetine, mirtazapine, tianeptine	
Cognitive impairment, dementia, Alzheimer’s disease		Desvenlafaxine, venlafaxine, duloxetine, sertraline, citalopram/escitalopram, bupropion, agomelatine, vortioxetine, mirtazapine	Nortriptyline
Pain disorders	Duloxetine, desvenlafaxine, venlafaxine	Nortriptyline	

### Strategies for Managing Inadequate Response and Treatment-Resistant Depression

**Figure 3:**
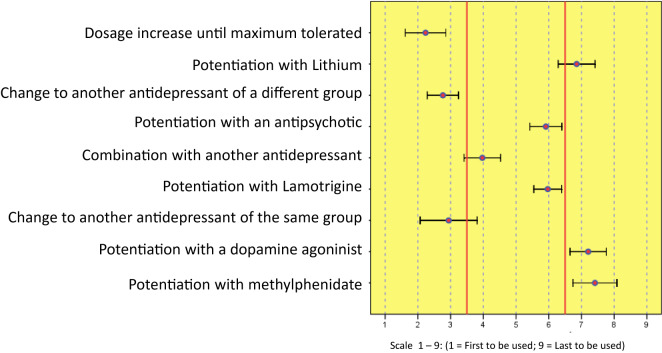
Priority strategy for efficacy in treatment-resistant depression.

**Figure 3** gathers the answers expressing priority actions when there is inadequate response to the initial antidepressant treatment, targeting efficacy.

**Figure 4:**
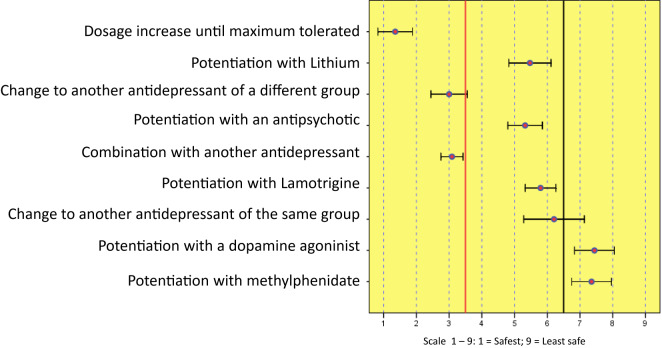
Priority strategy for safety in treatment-resistant depression.

**Figure 4** gathers the answers for priority actions when there is inadequate response to the initial antidepressant treatment, in terms of safety.

## Discussion

The discussion of results is approached using a question-answer format, since this makes understanding and grouping into homogeneous areas easier.

### Concepts, Clinical Aspects, and Diagnosis Process

A basic question is to consider whether geriatric depression as an entity is sufficiently defined in itself and in relation to that of the adult. Furthermore, it is important to discern which are the relevant aspects for the diagnostic process.

#### Are There any Differences Between the Clinical Symptoms of Depression in Adults and in the Elderly?

The panel of experts defended the importance of the clinical differences between depression in old age and depression in younger adults, specifically stressing a possible increase in anxiety, hypochondriac symptoms, suicidal ideation, and functional impact in the elderly, alongside with less likeliness to express sadness.

It should be noted that whether there are differences or not in symptomatology—somatic, cognitive, emotional—between depression in the elderly and in young individuals has been discussed at length (23), with authors and studies advocating one or the other position, although, in general, clinicians are inclined to acknowledge the existence of differences, which would allow greater diagnostic precision. Despite this, such differences are not contemplated in classification systems such as ICD-11 and DSM-5 (24).

#### Does Current Nosology Adequately Reflect Depression in the Elderly?

There is major consensus on considering that the criteria of current nosology to diagnose and classify depression in the latest stages of life are insufficient. According to our experts, the particularities of the clinical expression of depression in older adults, its evolution (which is associated with a greater risk for becoming chronic and with higher relapse rates) and its high comorbidity with other somatic and brain pathologies (25), are not adequately reflected in standard criteria.

The practically unanimous recommendation of our experts, which is consistent with that found in the scientific literature (26, 27), is the appropriateness of establishing certain specific criteria for depression in the elderly that may reflect its clinical and evolutionary particularities, thus enabling better understanding and easier identification of such clinical patterns and selection of the appropriate treatment.

It is not possible to infer from the previous statements that experts regard depression in the elderly as a different clinical entity from that in adults, but rather that they recommend greater alertness when gathering the clinical singularities in the diagnostic algorithm.

#### Are the Concepts of Late-Onset Depression, Vascular Depression and Depressive Pseudodementia Useful?

A favorable agreement on the clinical usefulness of the concepts of “late-onset depression” and “vascular depression” was reached, although there is not enough empirical evidence of them yet. In the first case, interest is associated with a tendency to the onset of more prominent cognitive symptoms, brain lesions – for example, of a vascular nature – and a higher risk for developing dementia in cases of late onset (28). As regards vascular depression, the presence of characteristic lesions detected through magnetic resonance imaging can help to shed light on certain cases of poor therapeutic response and higher risk for evolving into dementia (29).

On the other hand, there is no consensus on the clinical usefulness of the concept of “depressive pseudodementia”, a traditional term that refers to the onset of symptoms involving such prominent cognitive and functional impairment that they imitate the presence of a dementia disorder, but that are really caused by a case of depression and are therefore approachable using the appropriate antidepressant treatment. The classical controversies around the usefulness of the concept are reflected in the dispersion of answers obtained in this consensus. In any case, it must be noted that the term has plainly fallen into disuse in scientific literature.

#### Should the Process of Diagnosing Depression in the Elderly Include Routine Laboratory Tests and Neuroimaging?

The request for laboratory testing throughout the process of diagnosing geriatric depression has been a subject of discussion. According to our experts, the high comorbidity that is often present in these patients, many of them sufferers of multiple pathologies and also polymedicated, makes it necessary to carry out laboratory tests. Their opinion, however, is less unanimous as to the routine use of neuroimaging tests, despite their importance in the differential diagnosis of dementia cases (30). Nevertheless, this dispersion in answers could be nothing but the reflection of different styles and protocols in patient care, as well as of differences in the professional training or particularities of the work environment.

#### Is Screening of Depression in the Elderly Advisable? What Contexts Would Require it?

It is suspected that a substantial percentage of cases of depression in the elderly are not adequately identified, which would preclude appropriate care. A specific diagnostic category for geriatric depression would probably allow better identification of the process. To develop, implement and maintain an efficient detection process seems to be the first step towards a successful therapeutic approach.

We have gathered the opinion of experts on the suitability of using screening strategies at different levels (primary care, nursing homes for the elderly or through teleassistance services) for all senior patients. Most answers expressed agreement with the use of such strategies for the detection of these processes. However, there was a percentage (around 10%) who did not believe in the appropriateness of systematic screening for depression in elderly patients in primary care, an opinion that is in contrast with the efforts that are being made to assign initial treatment in depression cases in the first levels of care, regardless of the population group that is affected (31, 32).

Three interesting questions referred to risk factors were added to those concerning diagnosis:

#### Is Physical Illness a Greater Risk Factor for Suicide in Males?

There was consensus that the presence of physical conditions is a more significant risk factor for suicide in men than in women.

#### Do Older Adults who are not Depressed but Have Thoughts of Death Require Clinical Care?

There was consensus among the experts that the presence of thoughts of death in older adults who are apparently not depressed requires clinical care and assessment. This is because ideas of death in the elderly are associated with significant psychological distress, even in people who are not diagnosed with depression, and can be a symptom of subclinical depression or even a risk factor for suicide (33).

#### Is Disclosure of the Diagnosis of Dementia a Risk Factor for Depression?

As well as regarding the path from autonomy to dependence as a risk factor for depression in older adults, disclosure of a diagnosis of dementia was signaled by the experts as a significant risk factor for depression in this population. Nevertheless, it should be noted that there is growing evidence suggesting that the diagnosis of dementia can be safely communicated if done so in an appropriate way (34).

### Psychotic Depression

Psychotic depression (PD) is to date one of the most controversial clinical syndromes in the specific psychopathology of older adults, especially in aspects regarding its clinical presentation, therapeutics, and prognosis, and this consensus devotes a high number of questions to it.

#### Are There Nosologically Specific Symptoms That may Lead to a Diagnosis of PD in Elderly Patients?

At the psychopathological level, PD in general has been regarded by certain authors as a separate nosological entity. Psychotic symptoms are considered a criterion of endogeneity and a manifestation more commonly found in old age (35, 36). There is currently enough scientific and empirical evidence to support the diagnostic specificity of psychomotor disturbance (agitation or retardation or severe inhibition of motor response) in older adults with PD as opposed to younger adults (37, 38). There is no scientific evidence to support the nosological or diagnostic meaning of oppositional and negativistic behaviors in old age. The experts consulted failed to reach a consensus on the involvement of these behavioral symptoms in the diagnosis of psychotic depression in old age.

#### Does the Presence of Psychotic Symptoms in Older Adult Depression Confer an Increased Risk for Suicide or for the Development of Dementia?

There is sound scientific evidence of the risk for developing dementia associated with the cases of depression and psychosis in older adults (39, 40); however, there is not such support for the risk of psychotic depression developing into dementia as a specific nosological picture. This consensus shows an acceptable level of group agreement among experts that the onset of psychotic symptoms in geriatric depression confers an increased risk for evolution into dementia and is also related to a higher risk for suicide.

#### What is the Treatment of Choice for PD in Older Adults?

Most studies in PD in general note the superiority of electroconvulsive therapy (ECT) over combined antidepressant/antipsychotic (AD/AP) pharmacological therapy, and of the latter over pharmacological antidepressant monotherapy. There are scarce comparative studies on both therapeutic options. Current guidelines for the pharmacological treatment of depression in the elderly recommend the use of atypical antipsychotics, SSRI antidepressants, and dual-action antidepressants (41, 42). Over the years, ECT has been suggested as the first choice treatment by a large group of experts (14); however, in standard practice, most general psychiatrists do not follow such recommendation. One of the reasons for this is the progressive development of new psychiatric drugs with better tolerability and safety profiles for the elderly, which allows combined pharmacological therapy as the first option. On the other hand, the recommendations of widely used guidelines (43) on ECT suitability and uses suggest that this technique should only be the first choice in cases of severe functional impairment (need for fast action in cases of life-threatening depression, refractoriness to pharmacological treatment or impossibility of administering psychiatric drugs) regardless of age or psychiatric diagnosis. Clinical experience shows that most circumstances for which ECT is recommended as the first therapeutic option concur in a large percentage, if not in all, of the cases of elderly patients suffering from PD. However, in everyday clinical practice, its use as the first option remains unclear, with very different views based on each expert’s clinical experience and accessibility to the technique.

There is a high level of agreement among the experts consulted on the use of combined therapy with antipsychotics and antidepressants as the first therapeutic option in psychotic depression in the elderly. Likewise, there is broad consensus among them on the preference of using dual-action antidepressants over other pharmacological antidepressant options, and they also agree that ECT is the best therapeutic option when pharmacological treatment fails, although there is group disagreement as to its exclusive use in such circumstances. In the same line, there is no agreement among our experts on ECT being the first therapeutic option for PD in the elderly, so that its prescription remains subject to individual needs and to the risk-benefit balance of each case. After starting combined pharmacological treatment, and in absence of vital risk, no consensus is reached by the experts on the time required to assess refractoriness to pharmacological treatment prior to prescribing ECT, although the panel strongly disagrees with prescribing ECT beyond 24 weeks from the described situation, thus it should be used earlier.

#### Which is the Most Adequate Treatment to Prevent Relapse or Recurrence of PD in Older Adults?

Psychotic depression in the elderly is associated with high rates of relapse and/or recurrence, sometimes early in time (44, 45).

The group of experts reaches minimum group agreement on the recommendation of using maintenance ECT alongside combined AD/AP pharmacological maintenance therapy as the treatment of choice if ECT was needed, against therapeutic options such as pharmacological monotherapy or only maintenance ECT to prevent mid/long-term relapse and recurrence. The opinion of our experts is consistent with the results on long-term effectiveness that certain authors describe in the literature (46).

Adding lithium for maintenance treatment has been suggested as effective to prevent recurrence in recurrent depressive disorder with psychotic symptoms after its remission using ECT during the index episode (47) albeit there is more empirical than scientific evidence of this. The panel of experts globally disagreed on the choice of this therapeutic technique as part of maintenance treatment after good response to ECT in the acute phase.

### Depression and Dementia

There is a complex relationship between dementia and depression. This topic was extensively addressed in the first edition of this consensus (17), which is why some questions regarding certain basic items have not been repeated. The questions approached here are related to diagnostic criteria and treatment effectiveness.

Depression and dementia are very frequent disorders in the elderly population, and there is evidence that they share some common neurobiological mechanisms. Likewise, it seems well established that depression throughout life is a risk factor for dementia, and that late-onset depression can sometimes be a prodrome for a dementia syndrome (9, 48, 49). Nevertheless, since these are very common syndromes, comorbidities often occur, so that the clinician is confronted with unresolved issues.

#### Are the Diagnostic Criteria for Depression in Dementia Well Defined?

It should be emphasized that our experts’ answers show a high level of consensus on the questions regarding this issue. There is a clear consensus on the disagreement with the accuracy in the definition and usefulness of the current criteria for diagnosing depression in dementia/major neurocognitive disorder. The experts support the need to establish distinct criteria for depression in the different diseases or entities that might involve dementia/major neurocognitive disorder such as, for example, Alzheimer’s or Parkinson’s disease and also for the different severity phases of dementia, since their clinical expression may vary widely depending on the degree of impairment (50).

As regards to therapeutic interventions, the experts agree that both antidepressant drugs and psychological therapy can be effective in treating depression in dementia, although evidence in this area is still scarce and disparate (51).

### Pharmacological Treatment

The main therapeutic approach in geriatric depression consists on the use of antidepressant drugs, which is the most common first-line treatment. Despite the clinical importance of an adequate therapeutic approach to this condition, studies in this population group and the evidence they provide are still insufficient. Thus, evidence-based therapeutic recommendations in adult populations are often extrapolated to this age group, which might not be appropriate. Given its importance, this is the part that accounts for the highest number of questions of those addressed in this consensus.

#### Should Subclinical Depression be Treated Pharmacologically?

The experts consulted failed to reach an agreement on this issue, with a bimodal distribution where two clearly dichotomic opinions can be observed: one of the groups of panelists is against the use of antidepressant drugs, while the other chooses to use pharmacological treatment in subclinical depression.

#### Should Ancillary Tests be Performed Before Starting Treatment?

The experts were in favor of preforming laboratory tests and an electrocardiogram prior to choosing the appropriate antidepressant treatment. Their opinion is consistent with that found in the scientific literature regarding the advisable procedures before beginning treatment in geriatric depression (52).

#### What Are the First-line Pharmacological Groups in Geriatric Depression?

SSRI antidepressants were regarded as the first choice in the treatment of geriatric depression. More than half of the experts extended first-line treatment to dual-action antidepressants, although no statistical consensus was reached in this issue. By contrast, in the former edition of this consensus there was consensus that dual-action drugs are also first choice drugs (17).

#### Are There Differences Among the Different Antidepressant Groups?

The experts have agreed that dual-action antidepressants are more effective than SSRI in geriatric depression. This is in contrast with the fact that, as mentioned before, no agreement is reached on dual-action antidepressants being first-line drugs. This could be more of a reflection of clinical habits or economic criteria than of an application of the available clinical evidence although the latter is not unanimous (53–56). For example, studies focused on addressing depression in the presence of brain damage lead to a body of evidence regarding the lower efficacy of SSRI. Among other results, the findings reveal that the greater the damage to the white matter the lower the response to sertraline (57), also the greater effect of tricyclic antidepressants over SSRI in cases of stroke (58, 59) and the greater efficacy of nortriptyline and amitriptyline in depressive episodes in the context of Parkinson’s disease (60, 61).

In conclusion, although in the opinion of our experts SSRIs can be considered as first-choice agents, dual-action drugs are preferred in terms of efficacy.

#### Are Dietary Supplements (e.g. Omega-3) Effective in Geriatric Depression?

There was no consensus in favor or against this issue among the psychiatrists consulted, whose answers mainly reflect their lack of experience with these agents. While there are publications that support the use of dietary supplements in depressed elderly population (62), their use is not widespread, nor is there yet enough evidence to recommend their systematic use.

#### Is Response Latency Increased in Geriatric Depression?

There was consensus among the experts consulted on the presence of increased antidepressant response latency in older adults. This usually entails the need for a longer period to assess efficacy.

#### Does the Introduction of Antidepressants Increase the Risk for Suicide in Depressed Older Adults at the Beginning of Treatment?

This is a controversial issue regardless of age. Although there was a certain trend in favor of this assertion, experts did not reach consensus, providing very heterogeneous answers.

#### Do Older Adults Tolerate Antidepressants Worse Than Younger Adults?

No consensus was reached on the issue of different tolerability between dual-action antidepressants and SSRI. On the other hand, there was unanimous consensus that antidepressant drug-induced sexual dysfunction also happens and is a problem among the elderly population.

#### How Long Should Maintenance Treatment Last?

In the absence of scientific evidence on the issue, the scientific committee deemed it appropriate to ask the experts several questions about the right length of time for maintenance treatment and when it should be prolonged indefinitely.

It was agreed that one year would be a suitable length of time for maintenance therapy after an isolated episode, and there was disagreement on maintaining this treatment up to three or four years. Indefinite treatment was rejected for a first depressive episode, and the experts consulted agreed on recommending indefinite pharmacological treatment from the second depressive episode onwards.

#### Which Are the Antidepressants of Choice Based on Existing Medical Comorbidities?

As mentioned, the answers to this question, grouped according to the relevant condition, are gathered in **Table 11**. The broad range of drugs and clinical conditions examined make it impossible to generalize the findings. It can be highlighted that only 9 out of the 21 comorbidity situations proposed can be associated with a *specifically recommended* drug in terms of safety, agomelatine being the most frequently mentioned. Conversely, 14 of these situations note drugs *to be avoided* as unsafe or contraindicated in patients with such comorbidities, the tricyclic nortriptyline being the most repeated.

Desvenlafaxine and sertraline, followed by vortioxetine, are the antidepressants that appear most frequently as recommended or good alternatives.

#### Which Are the Most Recommended Strategies in Terms of Efficacy and Safety in Cases of Inadequate Response to Antidepressant Treatment?

Treatment-resistant depression is a significant concern among clinicians who care for depressed elderly patients. An international survey of 585 psychiatrists revealed that treatment-resistant depression could amount to between a quarter to a half of their workload, geriatric depression accounting for between 10 and 25% of this load (63). Although there are clinical trials and metanalyses that study strategies against resistant depression in adults, there are hardly any devoted to the elderly population, especially where the comparison of different strategies is concerned (64–67).

Panelists were provided with a list of possible strategies to use in the event of unsatisfactory response to a first prescribed antidepressant treatment, chosen among the most commonly mentioned in the literature (68), and were asked to arrange them in order of preference. We preferred to opt for a fairly open definition of inadequate response or resistance to treatment because several of them exist in the literature and each expert probably follows his or her personal approach. The indication given to the panelists was that they should order 9 common options in the form of progressive steps if the previous one was considered insufficient and deserved an action. The idea behind this procedure was to find an average of the progressive steps taken by the experts in order to generalize its usefulness.

Regarding the efficacy of the different options provided, the most widely supported first instance strategy was the escalation of the antidepressant to the maximum dose. This recommendation differs partly from the common procedure of using low doses in elderly patients and suggests increasing the dose to the maximum tolerated in cases of inadequate therapeutic response.

The second and third most supported strategies after this are changing to an antidepressant of a different group and changing to one belonging to the same group, respectively. It is estimated that these are the most common alternatives in any branch of medicine.

The answers suggested for cases when these strategies fail are of special interest. The combination of antidepressants in elderly patients with inadequate response is highly supported by the experts, followed by two possible potentiation actions using an antipsychotic or using lamotrigine, both of them with similar levels of support. Far below is lithium potentiation, and the potentiation with dopaminergic agents or methylphenidate comes last.

This information must be combined with that referred to safety aspects. The strategy that was indicated as the safest was the same that was chosen as the first alternative: dose escalation. This means that escalation to maximum dosage with modern antidepressants is believed to be not only effective, but also safe. Changing to an antidepressant of a different group or combining antidepressants is the next stated, with a similar degree of safety. On the following level, considered less safe, is improvement using an antipsychotic, lithium or lamotrigine -all three of them believed to be very similar in terms of safety-, and next, changing to an antidepressant of the same group. Improvement using dopamine agonists or methylphenidate are viewed as the least safe alternatives, which could perhaps reflect that experts do not have much experience with these agents.

In short, according to their efficacy and safety, the panel of experts recommends the following steps in order of preference for cases of resistance or insufficient response to antidepressant treatment:

1. Escalation to the maximum tolerated dose
2. Change of antidepressant
3. Combination with another antidepressant
4. Potentiation with an antipsychotic or with lamotrigine
5. Potentiation with lithium
6. Potentiation with dopamine agonists or methylphenidate

### Non-Pharmacological Biological Treatments (Fundamentally ECT)

There is currently broad agreement about the effectiveness of Electroconvulsive Therapy (ECT) in aged patients (69), especially in cases of severe or drug treatment-resistant major depression or when there is a physical condition that makes pharmacological treatment problematic. ECT is associated with an effectivity between 60 and 80% in the elderly population (70).

The incidence of side effects is low, and there are hardly any absolute contraindications, so that it is safe and especially indicated in the elderly (71), or very elderly population (72). Beyond such proven efficacy, other controversial questions were raised for assessment.

#### Is ECT Indicated in Vascular Depression?

The most common response was yes. Although the concept of vascular depression is still partially questioned, the answers could be directed towards the effectivity of ECT when vascular factors are clearly involved in cases of depression in older adults (73).

#### Is ECT Indicated in Patients Suffering From Depression and Dementia?

Here, most of the consensus group also answered affirmatively. Despite the fact that one of the potential side effects of ECT is secondary cognitive impairment, this is not considered decisive to discourage its use in severe depressive episodes suffered by patients with cognitive impairment or dementia. It is not uncommon for patients with dementia to experience regressive behaviors, such as active food intake refusal or other severe behavioral disturbances, such as agitation or aggression (74), that can involve serious short-term implications, so that, in such cases, ECT can totally or partially reverse the symptoms within a short period of time (75).

#### Should Bilateral ECT be Used Only When Unilateral ECT Does not Yield Satisfactory Results?

Most of the group disagrees with this assertion. There is evidence, and controversy, regarding the greater effectiveness of using bilateral over unilateral ECT in cases of severe depression (76). Reluctance to initially using bilateral ECT in depressed older adults is based on the purpose of avoiding cognitive effects in elderly patients, although, as with psychiatric drugs, the decision should always aim towards a more effective therapeutic response, balanced with a reasonable assessment of its side effects (77).

#### Do Cognitive Effects Associated With ECT Significantly Limit its Prescription in Geriatric Depression?

Group consensus is again reached against this assertion. ECT-related cognitive effects are frequent within all age ranges and are one of the common reasons for complaint among patients. Moreover, there are a series of concurrent factors in the elderly that are associated with greater cognitive side-effects: older age (78) pre-existing cognitive impairment, neurological diseases, such as basal ganglia lesions and hyperintensities on magnetic resonance imaging (79). In such situations, successful resolution of the severe depressive episode should prevail, even in old age, since there is no evidence of ECT *per se* causing brain damage (69) and this is what the consulted experts express.

#### Should Transcranial Magnetic Stimulation Be Taken Into Consideration as a Therapeutic Option In Resistant Depression in old age?

Transcranial magnetic stimulation (rTMS) consists in magnetic stimuli applied in a rhythmic and repeated way, with different frequency ranges. It has proven effective to treat depression and, also, chronic pain, stroke, Parkinson’s disease, schizophrenia, and obsessive-compulsive disorder among others (80).

The experts took no position for or against the statement, probably for a lack of direct experience or the absence of conclusive data on its effectiveness.

### Psychotherapeutic Treatments

There is not yet much consensus among experts on the issue of psychotherapy in older adults, perhaps due in part to the heterogeneity of the studies available and the difficulty to compare them.

#### Is Psychotherapy as Effective in the Elderly as in Adults?

The efficacy of psychological treatments for depression in older adults is already sufficiently supported in the literature. Meta-analytic studies involving randomized controlled trials (81), systematic reviews (82) and studies comparing pharmacotherapy and psychotherapy (83, 84) conclude that psychotherapeutic treatment is useful in addressing depression in older adults, with moderate to high effects and low heterogeneity. More recent studies (23, 85, 86) support the existing research, stressing that psychotherapies are an effective treatment for depression in old age, although the magnitude of the effect varies according to the type of control group.

All of the above endorses the fact that the elderly can benefit from psychotherapeutic treatments, when available. But, despite their acknowledged efficacy, their use on this segment of the population is still rare. This seems due to the scarce availability in healthcare services of these treatments for the elderly and to the prejudices of professionals who are generally reluctant to carry out these interventions (87, 88).

While old age in itself is not an obstacle to psychotherapy (89), in our consensus there is no homogeneous view on the matter, and a relatively high percentage of panelists (45.7%) believe that it is less effective in older adults.

Psychological treatments might be more relevant in the elderly, since aged patients with depression are more vulnerable to the side effects and pharmacological interactions of drugs. (11).

Our study shows general consensus in favor of psychotherapy’s efficacy in treating subclinical geriatric depression. The studies reviewed that included patients suffering from minor depression or dysthymia provided evidence of psychotherapy’s greater effect.

Research in the area suggests that psychotherapies are effective even in the presence of cognitive impairment (90) or executive dysfunction (91). The experts participating in this consensus agree with the idea that the presence of mild cognitive impairment/mild dementia does not limit the application of psychotherapy.

#### Is Psychotherapy as Effective as Pharmacological Treatments in Geriatric Depression?

Even though 45.7% of the experts consulted believe that psychotherapy is not as effective as pharmacological treatment in the elderly, the opinion is not homogeneous enough to achieve consensus.

Among the studies that exclusively compare psychotherapeutic treatment with treatment combining psychotherapy and pharmacotherapy (92), the combined choice is believed to be more effective than psychological treatment alone, placebo, pharmacotherapy alone or the combination of psychotherapy and placebo, although the relevance of this difference from a clinical perspective is unclear. Results suggest that the inclusion of psychotherapy reduces lack of response and helps toward patients’ adherence to treatment.

### Comorbidity and Preventive Aspects

#### Is Late-Onset Depression a Risk Factor for Medical Conditions?

Research confirms the vulnerability of depressed individuals to physical diseases in general. In 2004, the World Health Organization included comorbidity between depression and chronic physical illness among its ten world public health concerns.

The association between depression and medical conditions is bidirectional, being especially relevant in old age. While such relationship has been extensively studied, and although there is an increase in the number of studies where depression is regarded as a result of chronic conditions (93, 94), there is also evidence of the inverse relationship. According to longitudinal population-based studies, patients diagnosed with depression are at higher risk for certain medical conditions and for higher degrees of disability as compared to the general population (95). Related to functional condition and chronic clinical burden, older adults with a prior history of depression show faster rates of physical health decline than those who have never suffered from such disorder. This means that depression can be a risk marker for physical health deterioration (96). In prospective analyses, the probability of depressed patients to be affected by physical conditions during the follow-up period was almost two-thirds higher than in community controls (97).

In our study, there is outstanding consensus with regard to the comorbidity of geriatric depression. More than 90% of the experts agreed that the presence of late-onset depression makes it necessary to consider the eventual onset of a neurodegenerative disorder (e.g. dementia or Parkinson’s disease) or of serious medical conditions such as cancer or heart disease (77% of the group of experts).

#### What Weight Does Loneliness and Social Isolation Carry in the Prevention of Depression in Old Age?

There are robust data supporting that the presence of depression in the elderly population is associated with greater social isolation. Loneliness and social isolation are linked with higher rates of morbimortality, especially from mental disorders or cardiovascular disease (98). Older adults living with relatives or those who have higher levels of social contact score significantly higher in physical, mental, and emotional health, which means that these two factors have a protection effect against the onset of depressive symptoms (93).

In our study, the experts surveyed show maximum consensus on the assertion that interventions aimed at reducing social isolation are a significant strategy that is effective in the prevention of depression in older adults.

#### What Role Does Physical Exercise Play?

Sustained physical activity is associated with increased longevity and better maintenance of physical and mental functions (99). Physical exercise has a positive impact on mood and improves quality of life. Besides, there is evidence of it being effective in the treatment of depressed elderly patients, also improving their quality of life (100–102).

In our study, there is broad and consistent consensus among the experts on the significance of physical exercise as a protecting factor against depression in old age.

#### What Role Can Teleassistance Services Play?

According to the reviews on interventions to reduce suicide in the elderly population, the most effective were multifaceted depression detection and management programs in primary care, therapeutic approaches (pharmacotherapy and psychotherapy), community programs, and telephone counseling for vulnerable older adults (103, 104).

Our experts agree that teleassistance services are useful in reducing the risk for suicide in older adults.

#### Do Patients Living in Geriatric Institutions Receive the Same Type of Care as Those Living in the Community?

There was also consensus in our study that elderly people living in geriatric nursing homes do not have access to the same treatments (antidepressants, psychotherapy, ECT, etc.) as those living in the community. It seems evident that therapeutic resources vary widely according to place of residence and type of geriatric institution, which has an impact on both care and prevention.

### Professional Training to Address Depression in the Elderly

Addressing depression in elderly patients poses significant challenges for professionals. Its clinical characteristics could partly explain why it is an infradiagnosed disease. Clinical comorbidity (105), the presence of cognitive impairment, different phenomenology and clinical expressions, or frequent self-justification of the symptoms as due to poor health or accumulation of adversities make identification of depression in old age difficult (106). In cases of sub-syndromic depression, the problem of infradiagnosis is even greater (107).

#### Is the Training Currently Received by Medical Residents in Psychiatry Good Enough to Successfully Address Depressive Disorders in Older Adults?

The mostly negative answer to this question reflects the opinion that the training in geriatric psychiatry that is provided during the training of psychiatrists is insufficient. A survey reported that in Spain, around 60% of the psychiatry residents lack any type of specific training in psychogeriatrics, despite awareness (87.7%) of it being an especially relevant area of training. Psychiatry residents’ supervisors believe (61.9%) that the former are not provided with adequate training and consider (81%) that this should be a compulsory subject (108).

### Do General Psychiatrists Require Specific Training to Successfully Address Depressive Disorders in Older Adults?

The most common answer is yes. The detection of affective disorders in the elderly presents complexities that can only be addressed if psychiatrists have the adequate skills. Thus, low rates of identification of depressive symptomatology have been documented among psychiatrists (109). The need for training in geriatric psychiatry has been identified and requested on several occasions (110–112). More generally, there is evidence of deficiencies in the training in psychogeriatrics of psychiatrists (113).

#### Because of its Complexity, Should Depression in Older Adults Mainly be Treated by Psychiatrists?

While there are certain nuances, it is acknowledged that psychiatrists are the right specialists for the diagnosis and therapeutic management of depression in older adults, especially in the most severe cases, although many depressive episodes are currently addressed in primary care. The difficulties experienced by general practitioners in detecting geriatric depression have to do among other causes, with patients’ reasons for consulting—being the most common physical ailments (114)—with lack of familiarity with the use of diagnostic criteria and clinical assessment scales (115) and the greater presence of incomplete, mild, or sub-syndromic depressive disorders (116).

## Conclusions

In summary, a broad degree of consensus was achieved, reaching two thirds of the questions raised.

Clinical differences between depression in the elderly and in younger adults and the need for these differences to appear in current classification systems (including the concepts of late-onset and vascular depression) were highlighted. Specific criteria for depression in the context of dementia are also needed. Routine laboratory tests but not neuroimaging is recommended in the regular clinical assessment of geriatric depression.

The presence of late-onset depression makes it necessary to consider the subsequent onset of a neurodegenerative disorder such as dementia or Parkinson’s disease or of serious medical conditions such as cancer or heart disease.

The onset of psychotic symptoms in geriatric depression confers an increased risk for evolution into dementia and is also related to a higher risk for suicide. Combined therapy with antipsychotics and antidepressants is considered the first therapeutic option in psychotic depression in the elderly. ECT is the best therapeutic option when pharmacological treatment fails.

The experts were in favor of preforming laboratory tests and an electrocardiogram prior to choosing the appropriate pharmacological antidepressant treatment. Although SSRI antidepressants were regarded as the first choice, dual-action antidepressants were considered more effective than SSRI in geriatric depression.

Experts consulted agreed on the presence of an increased antidepressant response latency in older adults. Once response is achieved, one year is deemed necessary for maintenance therapy after an isolated episode in old age and recommendations are made for indefinite pharmacological treatment from the second depressive episode onwards.

In terms of pharmacological treatments, agomelatine was the most widely mentioned drug recommended in comorbid conditions in terms of safety. Desvenlafaxine, sertraline, and vortioxetine, were the most frequently recommended antidepressants in comorbid conditions in general.

Combining parameters of efficacy and safety, experts recommended to follow some gradual steps to address cases of treatment resistance. These are: 1. Escalation to the maximum tolerated dose; 2. Change of antidepressant; 3. Combination with another antidepressant; 4. Potentiation with an antipsychotic or with lamotrigine; 5. Potentiation with lithium; 6. Potentiation with a dopamine agonist or methylphenidate.

There is broad agreement about the effectiveness of ECT in elderly patients especially in cases of severe depression or in the presence of a significant physical condition. ECT can also be recommended in vascular depression and in severely depressed patients with dementia.

Although it might be less effective than in adults, the experts recommend the use of psychotherapy for clinical and subclinical geriatric depression even in the presence of mild cognitive impairment or mild dementia. Interventions aimed at reducing social isolation and promoting physical exercise are strategies that can be effective in the prevention of depression in older adults.

Psychiatrists are considered the adequate specialists for the diagnosis and therapeutic management of depression in older adults, especially in severe cases. Thus, all psychiatry medical residents should receive a specific training in geriatric psychiatry in general and in geriatric depression in particular

We hope that this consensus can contribute to reduce uncertainty in the diagnosis and treatment of elderly patients with depressive disorders, but further formal research is needed to shed light on the still unclear and controversial aspects of this disease

## Data Availability Statement

The datasets generated for this study are available on request to the corresponding author.

## Ethics Statement

Ethical approval and written, informed consent was not required according to local legislation and national guidelines.

## Author Contributions

All the authors contributed equally to the study design and manuscript preparation. LA-O coordinated the expert group.

## Funding

The Spanish Psychogeriatric Association received economic support from Pfizer Spain for the technical aspects of this study (Delphi analysis). Pfizer was not involved in the study design, collection, analysis, interpretation of data, the writing of this article or the decision to submit it for publication.

## Conflict of Interest

LA-O has received honoraria by Exeltis, Janssen, Lundbeck, Neuraxfarm, Otsuka, Servier and Pfizer. MM-C has received honoraria by Pfizer, Servier, and Lundbeck. MR-P has received honoraria by Janssen, Ludnbeck, Neuraxpharm, Otsuka, and Pfizer. MS-P has received honoraria by Exeltis, Lundbeck, Otsuka, Pfizer, and Servier.

The remaining authors declare that the research was conducted in the absence of any commercial or financial relationships that could be construed as a potential conflict of interest.

## References

[1] Wittchen HU Jacobi F Rehm J Gustavsson A Svensson M Jonsson B The size and burden of mental disorders and other disorders of the brain in Europe 2010. Eur Neuropsychopharmacol (2011) 21(9):655–79. 10.1016/j.euroneuro.2011.07.018 21896369

[2] Olivera J Benabarre S Lorente T Rodriguez M Pelegrin C Calvo JM Prevalence of psychiatric symptoms and mental disorders detected in primary care in an elderly Spanish population. The PSICOTARD Study: preliminary findings. Int J Geriatr Psychiatry (2008) 23(9):915–21. 10.1002/gps.2004 18311851

[3] Global Burden of Disease Study C Global, regional, and national incidence, prevalence, and years lived with disability for 301 acute and chronic diseases and injuries in 188 countries, 1990-2013: a systematic analysis for the Global Burden of Disease Study 2013. Lancet (2015) 386(9995):743–800. 10.1016/S0140-6736(15)60692-4 26063472 PMC4561509

[4] Valiengo Lda C Stella F Forlenza OV Mood disorders in the elderly: prevalence, functional impact, and management challenges. Neuropsychiatr Dis Treat (2016) 12:2105–14. 10.2147/NDT.S94643 PMC5003566 27601905

[5] Sjoberg L Karlsson B Atti AR Skoog I Fratiglioni L Wang HX Prevalence of depression: Comparisons of different depression definitions in population-based samples of older adults. J Affect Disord (2017) 221:123–31. 10.1016/j.jad.2017.06.011 28645024

[6] Diniz BS Lin CW Sibille E Tseng G Lotrich F Aizenstein HJ Circulating biosignatures of late-life depression (LLD): Towards a comprehensive, data-driven approach to understanding LLD pathophysiology. J Psychiatr Res (2016) 82:1–7. 10.1016/j.jpsychires.2016.07.006 27447786 PMC9344393

[7] Steffens DC Wang L Manning KJ Pearlson GD Negative Affectivity, Aging, and Depression: Results From the Neurobiology of Late-Life Depression (NBOLD) Study. Am J Geriatr Psychiatry (2017) 25(10):1135–49. 10.1016/j.jagp.2017.03.017 PMC5600659 28457805

[8] Palmer K Di Iulio F Varsi AE Gianni W Sancesario G Caltagirone C Neuropsychiatric predictors of progression from amnestic-mild cognitive impairment to Alzheimer’s disease: the role of depression and apathy. J Alzheimers Dis (2010) 20(1):175–83. 10.3233/JAD-2010-1352 20164594

[9] John A Patel U Rusted J Richards M Gaysina D Affective problems and decline in cognitive state in older adults: a systematic review and meta-analysis. Psychol Med (2019) 49(3):353–65. 10.1017/S0033291718001137 PMC6331688 29792244

[10] Avasthi A Grover S Clinical Practice Guidelines for Management of Depression in Elderly. Indian J Psychiatry (2018) 60(Suppl. 3):S341–S62. 10.4103/0019-5545.224474 PMC5840909 29535469

[11] MacQueen GM Frey BN Ismail Z Jaworska N Steiner M Lieshout RJ Canadian Network for Mood and Anxiety Treatments (CANMAT) 2016 Clinical Guidelines for the Management of Adults with Major Depressive Disorder: Section 6. Special Populations: Youth, Women, and the Elderly. Can J Psychiatry (2016) 61(9):588–603. 10.1177/0706743716659276 27486149 PMC4994788

[12] Canadian Agency for Drugs and Technologies in Health Diagnosing, Screening, and Monitoring Depression in the Elderly: A Review of Guidelines. In: CADTH Rapid Response Reports. Ottawa (ON): CADTH (2015). 26468558

[13] Peck S Clinical guideline for the care and treatment of older people with depression in a general hospital setting: Isle of Wight Healthcare. (2003). Available from: http://www.iow.nhs.uk/Department/mental/older/pdfs/Depression%20Guideline.pdf.

[14] Baldwin RC Chiu E Katona C Graham N Guidelines on depression in older people: practicing the evidence. London: Martin Dunitz Ltd. (2002).

[15] Lebowitz BD Pearson JL Schneider LS Reynolds CF 3rd GS A ML B Diagnosis and treatment of depression in late life. Consensus statement update. JAMA (1997) 278(14):1186–90. 10.1001/jama.278.14.1186 9326481

[16] Canadian Coalition for Seniors’ Mental Health The Assessment and Treatment of Depression. Toronto: CCSMH (2006).

[17] Martin-Carrasco M Aguera-Ortiz L Caballero-Martinez L Cervilla-Ballesteros J Menchon-Magrina JM Montejo-Gonzalez AL Consensus of the SEPG on depression in the elderly. Actas Esp Psiquiatr (2011) 39(1):20–31. 21274819

[18] Goodman LA Snowball Sampling. Ann Math Stat (1961) 32:148–70. 10.1214/aoms/1177705148

[19] Boulkedid R Abdoul H Loustau M Sibony O Alberti C Using and reporting the Delphi method for selecting healthcare quality indicators: a systematic review. PloS One (2011) 6(6):e20476. 10.1371/journal.pone.0020476 21694759 PMC3111406

[20] Hutchings A Raine R Sanderson C Black N A comparison of formal consensus methods used for developing clinical guidelines. J Health Serv Res Policy (2006) 11(4):218–24. 10.1258/135581906778476553 17018195

[21] Custer RL Scarcella JA Stewart BR The modified Delphi technique: A rotational modification. J Vocational Tech Educ (1999) 15(2):1–10. 10.21061/jcte.v15i2.702

[22] Dalkey NC The Delphi Method: an experimental study of group opinion. Santa Mónica (California): The Rand Corporation (1969).

[23] Haigh EAP Bogucki OE Sigmon ST Blazer DG Depression Among Older Adults: A 20-Year Update on Five Common Myths and Misconceptions. Am J Geriatr Psychiatry (2018) 26(1):107–22. 10.1016/j.jagp.2017.06.011 28735658

[24] Power C Reene E Lawlor BA Depression in Late Life. Etiology, Presentation, and Management. In: Chiu H Shulman K, editors. Mental Health and Illness of the Elderly Mental Health and Illness Worldwide. Singapur: Springer (2017). p. 187–218.

[25] Ismail Z Fischer C McCall WV What characterizes late-life depression? Psychiatr Clin North Am (2013) 36(4):483–96. 10.1016/j.psc.2013.08.010 24229652

[26] Belvederi Murri M Amore M Respino M Alexopoulos GS The symptom network structure of depressive symptoms in late-life: Results from a European population study. Mol Psychiatry (2018). 10.1038/s41380-018-0232-0 30171210

[27] Cervilla Ballesteros J Sindromes depresivos. In: Agüera Ortiz L Martin Carrasco M Cervilla Ballesteros J, editors. Psiquiatría Geriatrica. Barcelona: Elsevier (2006). p. 409–25.

[28] Grayson L Thomas A A systematic review comparing clinical features in early age at onset and late age at onset late-life depression. J Affect Disord (2013) 150(2):161–70. 10.1016/j.jad.2013.03.021 23623421

[29] Aizenstein HJ Baskys A Boldrini M Butters MA Diniz BS Jaiswal MK Vascular depression consensus report - a critical update. BMC Med (2016) 14(1):161. 10.1186/s12916-016-0720-5 27806704 PMC5093970

[30] Leyhe T Reynolds CF 3rd Melcher T Linnemann C Kloppel S Blennow K A common challenge in older adults: Classification, overlap, and therapy of depression and dementia. Alzheimers Dement (2017) 13(1):59–71. 10.1016/j.jalz.2016.08.007 27693188

[31] Smithson S Pignone MP Screening Adults for Depression in Primary Care. Med Clin North Am (2017) 101(4):807–21. 10.1016/j.mcna.2017.03.010 28577628

[32] Hall CA Reynolds-Iii CF Late-life depression in the primary care setting: challenges, collaborative care, and prevention. Maturitas. (2014) 79(2):147–52. 10.1016/j.maturitas.2014.05.026 PMC4169311 24996484

[33] Joo J Hwang S Gallo JJ Death Ideation and Suicidal Ideation in a Community Sample Who Do Not Meet Criteria for Major Depression. Crisis. (2016) 37(2):161–5. 10.1027/0227-5910/a000365 PMC5116433 27232430

[34] Goldfarb D Sheard S Shaughnessy L Atri A Disclosure of Alzheimer’s Disease and Dementia: Patient- and Care Partner-Centric Decision-Making and Communication. J Clin Psychiatry (2019) 80(2). 10.4088/JCP.MS18002BR1C 30900850

[35] Parker G Hadzi-Pavlovic D Hickie I Boyce P Mitchell P Wilhelm K Distinguishing psychotic and non-psychotic melancholia. J Affect Disord (1991) 22(3):135–48. 10.1016/0165-0327(91)90047-V 1918657

[36] Gournellis R Oulis P Howard R Psychotic major depression in older people: a systematic review. Int J Geriatr Psychiatry (2014) 29(8):789–96. 10.1002/gps.4065 25191689

[37] Cuevas-Esteban J Iglesias-Gonzalez M Rubio-Valera M Serra-Mestres J Serrano-Blanco A Baladon L Prevalence and characteristics of catatonia on admission to an acute geriatric psychiatry ward. Prog Neuropsychopharmacol Biol Psychiatry (2017) 78:27–33. 10.1016/j.pnpbp.2017.05.013 28533149

[38] Hyett MP Parker GB Proudfoot J Fletcher K Examining age effects on prototypic melancholic symptoms as a strategy for refining definition of melancholia. J Affect Disord (2008) 109(1-2):193–7. 10.1016/j.jad.2007.11.005 18162189

[39] Almeida OP Hankey GJ Yeap BB Golledge J Flicker L Depression as a risk factor for cognitive impairment in later life: the Health In Men cohort study. Int J Geriatr Psychiatry (2016) 31(4):412–20. 10.1002/gps.4347 26280254

[40] Almeida OP Ford AH Hankey GJ Yeap BB Golledge J Flicker L Risk of dementia associated with psychotic disorders in later life: the health in men study (HIMS). Psychol Med (2019) 49(2):232–42. 10.1017/S003329171800065X 29564993

[41] Alexopoulos GS Pharmacotherapy for late-life depression. J Clin Psychiatry (2011) 72(1):e04. 10.4088/JCP.7085tx2cj 21272511 PMC3314291

[42] Agüera Ortiz L Moríñigo A Olivera J Plá J Azanza Perea JR Documento de la Sociedad Española de Psicogeriatría sobre el uso de antipsicóticos en personas de edad avanzada. Psicogeriatría. (2017) 7 1:S1–S37.

[43] American Psychiatric Association Practice guideline for the treatment of patients with major depressive disorder (revision). American Psychiatric Association. Am J Psychiatry (2000) 157(4 Suppl.):1–45. 10767867

[44] Flint AJ Rifat SL Two-year outcome of psychotic depression in late life. Am J Psychiatry (1998) 155(2):178–83. 10.1176/ajp.155.2.178 9464195

[45] van Schaik AM Comijs HC Sonnenberg CM Beekman AT Sienaert P Stek ML Efficacy and safety of continuation and maintenance electroconvulsive therapy in depressed elderly patients: a systematic review. Am J Geriatr Psychiatry (2012) 20(1):5–17. 10.1097/JGP.0b013e31820dcbf9 22183009

[46] Navarro V Gasto C Torres X Masana G Penades R Guarch J Continuation/maintenance treatment with nortriptyline versus combined nortriptyline and ECT in late-life psychotic depression: a two-year randomized study. Am J Geriatr Psychiatry (2008) 16(6):498–505. 10.1097/JGP.0b013e318170a6fa 18515694

[47] Sackeim HA Haskett RF Mulsant BH Thase ME Mann JJ Pettinati HM Continuation pharmacotherapy in the prevention of relapse following electroconvulsive therapy: a randomized controlled trial. JAMA (2001) 285(10):1299–307. 10.1001/jama.285.10.1299 11255384

[48] Bennett S Thomas AJ Depression and dementia: cause, consequence or coincidence? Maturitas. (2014) 79(2):184–90. 10.1016/j.maturitas.2014.05.009 24931304

[49] Steffens DC Late-Life Depression and the Prodromes of Dementia. JAMA Psychiatry (2017) 74(7):673–4. 10.1001/jamapsychiatry.2017.0658 28514459

[50] Asmer MS Kirkham J Newton H Ismail Z Elbayoumi H Leung RH Meta-Analysis of the Prevalence of Major Depressive Disorder Among Older Adults With Dementia. J Clin Psychiatry (2018) 79(5). 10.4088/JCP.17r11772 30085437

[51] Reynolds CF 3rd Lenze E Mulsant BH Assessment and treatment of major depression in older adults. Handb Clin Neurol (2019) 167:429–35. 10.1016/B978-0-12-804766-8.00023-6 31753147

[52] Taylor WD Clinical practice. Depression in the elderly. N Engl J Med (2014) 371(13):1228–36. 10.1056/NEJMcp1402180 25251617

[53] Mukai Y Tampi RR Treatment of depression in the elderly: a review of the recent literature on the efficacy of single- versus dual-action antidepressants. Clin Ther (2009) 31(5):945–61. 10.1016/j.clinthera.2009.05.016 19539096

[54] Mulsant BH Blumberger DM Ismail Z Rabheru K Rapoport MJ A systematic approach to pharmacotherapy for geriatric major depression. Clin Geriatr Med (2014) 30(3):517–34. 10.1016/j.cger.2014.05.002 PMC4122285 25037293

[55] Thorlund K Druyts E Wu P Balijepalli C Keohane D Mills E Comparative efficacy and safety of selective serotonin reuptake inhibitors and serotonin-norepinephrine reuptake inhibitors in older adults: a network meta-analysis. J Am Geriatr Soc (2015) 63(5):1002–9. 10.1111/jgs.13395 25945410

[56] Kok RM Reynolds CF 3rd Management of Depression in Older Adults: A Review. JAMA (2017) 317(20):2114–22. 10.1001/jama.2017.5706 28535241

[57] Sheline YI Pieper CF Barch DM Welsh-Bohmer K McKinstry RC MacFall JR Support for the vascular depression hypothesis in late-life depression: results of a 2-site, prospective, antidepressant treatment trial. Arch Gen Psychiatry (2010) 67(3):277–85. 10.1001/archgenpsychiatry.2009.204 PMC2838210 20194828

[58] Price A Rayner L Okon-Rocha E Evans A Valsraj K Higginson IJ Antidepressants for the treatment of depression in neurological disorders: a systematic review and meta-analysis of randomised controlled trials. J Neurol Neurosurg Psychiatry (2011) 82(8):914–23. 10.1136/jnnp.2010.230862 21558287

[59] Robinson RG Schultz SK Castillo C Kopel T Kosier JT Newman RM Nortriptyline versus fluoxetine in the treatment of depression and in short-term recovery after stroke: a placebo-controlled, double-blind study. Am J Psychiatry (2000) 157(3):351–9. 10.1176/appi.ajp.157.3.351 10698809

[60] Costa FH Rosso AL Maultasch H Nicaretta DH Vincent MB Depression in Parkinson’s disease: diagnosis and treatment. Arq Neuropsiquiatr (2012) 70(8):617–20. 10.1590/S0004-282X2012000800011 22899034

[61] Menza M Dobkin RD Marin H Mark MH Gara M Buyske S A controlled trial of antidepressants in patients with Parkinson disease and depression. Neurology (2009) 72(10):886–92. 10.1212/01.wnl.0000336340.89821.b3 PMC2677475 19092112

[62] Varteresian T Lavretsky H Natural products and supplements for geriatric depression and cognitive disorders: an evaluation of the research. Curr Psychiatry Rep (2014) 16(8):456. 10.1007/s11920-014-0456-x 24912606 PMC4110105

[63] Arandjelovic K Eyre HA Lavretsky H Clinicians’ Views on Treatment-Resistant Depression: 2016 Survey Reports. Am J Geriatr Psychiatry (2016) 24(10):913–7. 10.1016/j.jagp.2016.05.010 PMC5540329 27591914

[64] Cooper C Katona C Lyketsos K Blazer D Brodaty H Rabins P A systematic review of treatments for refractory depression in older people. Am J Psychiatry (2011) 168(7):681–8. 10.1176/appi.ajp.2011.10081165 21454919

[65] Unutzer J Park M Older adults with severe, treatment-resistant depression. JAMA. (2012) 308(9):909–18. 10.1001/2012.jama.10690 PMC4136434 22948701

[66] Knochel C Alves G Friedrichs B Schneider B Schmidt-Rechau A Wenzler S Treatment-resistant Late-life Depression: Challenges and Perspectives. Curr Neuropharmacol (2015) 13(5):577–91. 10.2174/1570159X1305151013200032 PMC4761630 26467408

[67] Maust DT Oslin DW Thase ME Going beyond antidepressant monotherapy for incomplete response in nonpsychotic late-life depression: a critical review. Am J Geriatr Psychiatry (2013) 21(10):973–86. 10.1016/j.jagp.2013.01.030 PMC3543487 23567381

[68] Cristancho P Lenard E Lenze EJ Miller JP Brown PJ Roose SP Optimizing Outcomes of Treatment-Resistant Depression in Older Adults (OPTIMUM): Study Design and Treatment Characteristics of the First 396 Participants Randomized. Am J Geriatr Psychiatry (2019) 27(10):1138–52. 10.1016/j.jagp.2019.04.005 31147244

[69] Bernardo M González-Pinto A Urretavizcaya M Consenso Español sobre la terapia electroconvulsiva. Madrid: Consenso Español sobre la terapia electroconvulsiva (2018).

[70] Spaans HP Sienaert P Bouckaert F van den Berg JF Verwijk E Kho KH Speed of remission in elderly patients with depression: electroconvulsive therapy v. medication. Br J Psychiatry (2015) 206(1):67–71. 10.1192/bjp.bp.114.148213 25323140

[71] Geduldig ET Kellner CH Electroconvulsive Therapy in the Elderly: New Findings in Geriatric Depression. Curr Psychiatry Rep (2016) 18(4):40. 10.1007/s11920-016-0674-5 26909702

[72] Tew JD Jr. Mulsant BH Haskett RF Prudic J Thase ME Crowe RR Acute efficacy of ECT in the treatment of major depression in the old-old. Am J Psychiatry (1999) 156(12):1865–70. 10.1176/ajp.156.12.1865 10588398

[73] Currier MB Murray GB Welch CC Electroconvulsive therapy for post-stroke depressed geriatric patients. J Neuropsychiatr Clin Neurosci (1992) 4(2):140–4. 10.1176/jnp.4.2.140 1627974

[74] Acharya D Harper DG Achtyes ED Seiner SJ Mahdasian JA Nykamp LJ Safety and utility of acute electroconvulsive therapy for agitation and aggression in dementia. Int J Geriatr Psychiatry (2015) 30(3):265–73. 10.1002/gps.4137 PMC4524287 24838521

[75] Glass OM Forester BP Hermida AP Electroconvulsive therapy (ECT) for treating agitation in dementia (major neurocognitive disorder) - a promising option. Int Psychogeriatr. (2017) 29(5):717–26. 10.1017/S1041610216002258 28095946

[76] Pettinati HM Mathisen KS Rosenberg J Lynch JF Meta-Analytical Approach to Reconciling Discrepancies in Efficacy between Bilateral and Unilateral Electroconvulsive Therapy. Convuls Ther (1986) 2(1):7–17. 11940840

[77] Kellner CH Knapp R Husain MM Rasmussen K Sampson S Cullum M Bifrontal, bitemporal and right unilateral electrode placement in ECT: randomised trial. Br J Psychiatry (2010) 196(3):226–34. 10.1192/bjp.bp.109.066183 PMC2830057 20194546

[78] Zervas IM Calev A Jandorf L Schwartz J Gaudino E Tubi N Age-Dependent Effects of Electroconvulsive Therapy on Memory. Convuls Ther (1993) 9(1):39–42. 11941190

[79] Rami L Bernardo M Boget T Ferrer J Portella MJ Gil-Verona JA Cognitive status of psychiatric patients under maintenance electroconvulsive therapy: a one-year longitudinal study. J Neuropsychiatr Clin Neurosci (2004) 16(4):465–71. 10.1176/jnp.16.4.465 15616173

[80] Cristancho MA Helmer A Connolly R Cristancho P O’Reardon JP Transcranial magnetic stimulation maintenance as a substitute for maintenance electroconvulsive therapy: a case series. J ECT. (2013) 29(2):106–8. 10.1097/YCT.0b013e31827a70ba PMC3664096 23519219

[81] Cuijpers P van Straten A Smit F Psychological treatment of late-life depression: a meta-analysis of randomized controlled trials. Int J Geriatr Psychiatry (2006) 21(12):1139–49. 10.1002/gps.1620 16955421

[82] Wilson KC Mottram PG Vassilas CA Psychotherapeutic treatments for older depressed people. Cochrane Database Syst Rev (2008) 23(1):CD004853. 10.1002/14651858.CD004853.pub2 18254062

[83] Pinquart M Duberstein PR Lyness JM Treatments for later-life depressive conditions: a meta-analytic comparison of pharmacotherapy and psychotherapy. Am J Psychiatry (2006) 163(9):1493–501. 10.1176/ajp.2006.163.9.1493 16946172

[84] Parikh SV Segal ZV Grigoriadis S Ravindran AV Kennedy SH Lam RW Canadian Network for Mood and Anxiety Treatments (CANMAT) clinical guidelines for the management of major depressive disorder in adults. II. Psychotherapy alone or in combination with antidepressant medication. J Affect Disord (2009) 117(Suppl. 1):S15–25. 10.1016/j.jad.2009.06.042 19682749

[85] Huang AX Delucchi K Dunn LB Nelson JC A systematic review and meta-analysis of psychotherapy for late-life depression. Am J Geriatr Psychiatry (2015) 23(3):261–73. 10.1016/j.jagp.2014.04.003 24856580

[86] Kirkham JG Choi N Seitz DP Meta-analysis of problem solving therapy for the treatment of major depressive disorder in older adults. Int J Geriatr Psychiatry (2016) 31(5):526–35. 10.1002/gps.4358 26437368

[87] Wei W Sambamoorthi U Olfson M Walkup JT Crystal S Use of psychotherapy for depression in older adults. Am J Psychiatry (2005) 162(4):711–7. 10.1176/appi.ajp.162.4.711 PMC2921918 15800143

[88] Claver-Martín M Psicoterapia en el anciano. Psicogeriatría (2009) 1(3):175–8.

[89] Frazer CJ Christensen H Griffiths KM Effectiveness of treatments for depression in older people. Med J Aust (2005) 182(12):627–32. 10.5694/j.1326-5377.2005.tb06849.x 15963019

[90] Miller MD Reynolds CF.3rd Expanding the usefulness of Interpersonal Psychotherapy (IPT) for depressed elders with co-morbid cognitive impairment. Int J Geriatr Psychiatry (2007) 22(2):101–5. 10.1002/gps.1699 17096459

[91] Alexopoulos GS Raue PJ Kiosses DN Mackin RS Kanellopoulos D McCulloch C Problem-solving therapy and supportive therapy in older adults with major depression and executive dysfunction: effect on disability. Arch Gen Psychiatry (2011) 68(1):33–41. 10.1001/archgenpsychiatry.2010.177 21199963 PMC3018861

[92] Cuijpers P De Wit L Weitz E The combination of psychotherapy and pharmacotherapy in the treatment of adult depression: a comprehensive meta-analysis. J Evid Based Psychother. (2015) 15:147.

[93] Portellano-Ortiz C Garre-Olmo J Calvo-Perxas L Conde-Sala JL Depression and associated variables in people over 50 years in Spain. Rev Psiquiatr Salud Ment (2018) 11(4):216–26. 10.1016/j.rpsmen.2016.10.002 27939026

[94] Deschenes SS Burns RJ Schmitz N Associations between depression, chronic physical health conditions, and disability in a community sample: A focus on the persistence of depression. J Affect Disord (2015) 179:6–13. 10.1016/j.jad.2015.03.020 25841076

[95] Giner J Saiz Ruiz J Bobes J Zamorano E Lopez F Hernando T Spanish consensus on the physical health of patients with depressive disorders. Rev Psiquiatria Salud Ment (2014) 7(4):195–207. 10.1016/j.rpsmen.2014.10.003 25087131

[96] Cho HJ Lavretsky H Olmstead R Levin M Oxman MN Irwin MR Prior depression history and deterioration of physical health in community-dwelling older adults–a prospective cohort study. Am J Geriatr Psychiatry (2010) 18(5):442–51. 10.1097/JGP.0b013e3181ca3a2d PMC2860010 20220581

[97] Holahan CJ Pahl SA Cronkite RC Holahan CK North RJ Moos RH Depression and vulnerability to incident physical illness across 10 years. J Affect Disord (2010) 123(1-3):222–9. 10.1016/j.jad.2009.10.006 19880190

[98] Gene-Badia J Comice P Belchin A Erdozain MA Caliz L Torres S Profiles of loneliness and social isolation in urban population. Aten Primaria (2020) 52(4):224–32. 10.1016/j.aprim.2018.09.012 PMC7118570 30770152

[99] de Hoyos Alonso MDC Gorronogoitia Iturbe A Martin Lesende I Baena Diez JM Lopez-Torres Hidalgo J Magan Tapia P Actividades preventivas en los mayores. Actualización PAPPS 2018. Aten Primaria (2018) 50(Suppl. 1):109–24. 10.1016/s0212-6567(18)30365-2 PMC6836920 29866352

[100] Dauwan M Begemann MJH Slot MIE Lee EHM Scheltens P Sommer IEC Physical exercise improves quality of life, depressive symptoms, and cognition across chronic brain disorders: a transdiagnostic systematic review and meta-analysis of randomized controlled trials. J Neurol (2019). 10.1007/s00415-019-09493-9 PMC7990819 31414194

[101] Seo JY Chao YY Effects of Exercise Interventions on Depressive Symptoms Among Community-Dwelling Older Adults in the United States: A Systematic Review. J Gerontol Nurs. (2018) 44(3):31–8. 10.3928/00989134-20171024-01 29077980

[102] Schuch FB Vancampfort D Richards J Rosenbaum S Ward PB Stubbs B Exercise as a treatment for depression: A meta-analysis adjusting for publication bias. J Psychiatr Res (2016) 77:42–51. 10.1016/j.jpsychires.2016.02.023 26978184

[103] Okolie C Dennis M Simon Thomas E John A A systematic review of interventions to prevent suicidal behaviors and reduce suicidal ideation in older people. Int Psychogeriatr. (2017) 29(11):1801–24. 10.1017/S1041610217001430 28766474

[104] Lapierre S Erlangsen A Waern M De Leo D Oyama H Scocco P A systematic review of elderly suicide prevention programs. Crisis (2011) 32(2):88–98. 10.1027/0227-5910/a000076 21602163 PMC3728773

[105] Weinberger MI Roth AJ Nelson CJ Untangling the complexities of depression diagnosis in older cancer patients. Oncologist. (2009) 14(1):60–6. 10.1634/theoncologist.2008-0147 PMC3051280 19144682

[106] Burroughs H Lovell K Morley M Baldwin R Burns A Chew-Graham C ‘Justifiable depression’: how primary care professionals and patients view late-life depression? A qualitative study. Fam Pract (2006) 23(3):369–77. 10.1093/fampra/cmi115 16476699

[107] VanItallie TB Subsyndromal depression in the elderly: underdiagnosed and undertreated. Metabolism (2005) 54(5 Suppl. 1):39–44. 10.1016/j.metabol.2005.01.012 15877312

[108] Cuevas-Esteban J Baladón L Rodríguez MJ Fernández A Fusté-Boadella M Díaz D Formación en psicogeriatría en España: opinión de tutores y residentes de psiquiatría. Psicogeriatria (2012) 4(1):39–49.

[109] Brühl KG ¿Qué capacidad tienen los enfermeros y los auxiliares de enfermería para reconocer y tratar la depresión en las personas mayores? Informaciones Psiquiátricas (2009) 195:196.

[110] Camus V Katona C de Mendonca Lima CA Abdel-Hakam AM Graham N Baldwin R Teaching and training in old age psychiatry: a general survey of the World Psychiatric Association member societies. Int J Geriatr Psychiatry (2003) 18(8):694–9. 10.1002/gps.900 12891636

[111] World Health Organization Education in psychiatry of the elderly: a technical consensus statement. Geneva: WHO (1998).

[112] Gustafson L Burns A Katona C Bertolote JM Camus V Copeland JR Skill-based objectives for specialist training in old age psychiatry. Int J Geriatr Psychiatry (2003) 18(8):686–93. 10.1002/gps.899 12891635

[113] Pelegrín-Valero C La formación en psicogeriatría en nuestro país. Psicogeriatría (2009) 1:65–6.

[114] Mitchell AJ Rao S Vaze A Do primary care physicians have particular difficulty identifying late-life depression? A meta-analysis stratified by age. Psychother Psychosom (2010) 79(5):285–94. 10.1159/000318295 20616623

[115] Schwarzbach M Luppa M Hansen H Konig HH Gensichen J Petersen JJ A comparison of GP and GDS diagnosis of depression in late life among multimorbid patients - results of the MultiCare study. J Affect Disord (2014) 168:276–83. 10.1016/j.jad.2014.06.020 25080391

[116] MacDonald AJ Do general practitioners “miss” depression in elderly patients? Br Med J (Clin Res Ed). (1986) 292(6532):1365–7. 10.1136/bmj.292.6532.1365 PMC1340373 3085850

