# Supplementary material for: Depression in the Elderly. Consensus Statement of the Spanish Psychogeriatric Association
Source: Front Psychiatry. 2020 May 20;11:380. doi: 10.3389/fpsyt.2020.00380 (PMC7251154; doi:10.3389/fpsyt.2020.00380)

Topic Area 1.- Concepts, clinical aspects and risk factors

|  | **Consensus Disagreement** | **No consensus** | **Consensus Agreement** |
| --- | --- | --- | --- |
| 1.1. Late-onset depression (first episode in old age rather than in adulthood) is a clinically useful concept in psychiatry. |  |  | ◼ |
| 1.2. Vascular depression is a clinically useful concept in psychiatry. |  |  | ◼ |
| 1.3. Depressive pseudodementia is a clinically useful concept in psychiatry. |  | ◼ |  |
| 1.4. Depression in the elderly, as compared to adults, is more specifically associated with higher levels of anxiety. |  |  | ◼ |
| 1.5. Depression in the elderly, as compared to adults, is more specifically associated with higher levels of hypochondriac symptomatology. |  |  | ◼ |
| 1.6. Depression in the elderly, as compared to adults, is more specifically associated with less likelihood to express sadness. |  |  | ◼ |
| 1.7. Depression in the elderly, as compared to adults, is more specifically associated with higher levels of suicidal ideation. |  |  | ◼ |
| 1.8. Depression in the elderly, as compared to adults, is more specifically associated with greater impact on daily life. |  |  | ◼ |
| 1.9. Frailty states in the transition from autonomy to dependence entail a significant risk factor for depression in old age. |  |  | ◼ |
| 1.10. Disclosure of a dementia diagnosis is a significant risk factor for depression in old age. |  |  | ◼ |
| 1.11. Severe physical diseases in older adults are a higher risk factor for suicide in men than in women. |  |  | ◼ |
| 1.12. Non-depressed older adults with thoughts of death require clinical care. |  |  | ◼ |

Topic Area 1.- Concepts, clinical aspects and risk factors. Degree of agreement analysis


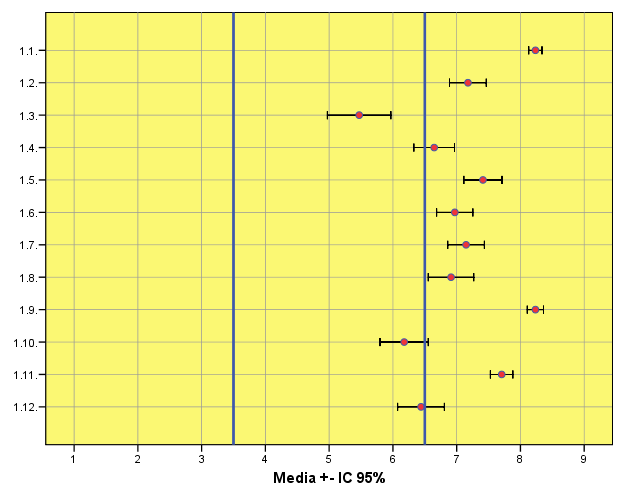


Topic Area 2.- Screening and diagnosis

|  | **Consensus Disagreement** | **No consensus** | **Consensus Agreement** |
| --- | --- | --- | --- |
| 2.1. The diagnostic criteria of standard nosologies (ICD 11, DSM5) are adequate to diagnose and classify depressive disorders in the elderly. | ◼ |  |  |
| 2.2. Specific diagnostic criteria for depressive disorders in the elderly population are necessary. |  |  | ◼ |
| 2.3. Teleassistance services should include some type of screening system for depression. |  |  | ◼ |
| 2.4. ALL elderly patients living in nursing homes should be systematically screened for depression. |  |  | ◼ |
| 2.5. ALL elderly patients should be systematically screened for depression in Primary Care. |  |  | ◼ |
| 2.6. Any diagnostic process in late-onset depression  should include laboratory tests. |  |  | ◼ |
| 2.7. Any diagnostic process in late-onset depression should include neuroimaging testing. |  | ◼ |  |

Topic Area 2.- Screening and diagnosis. Degree of agreement analysis


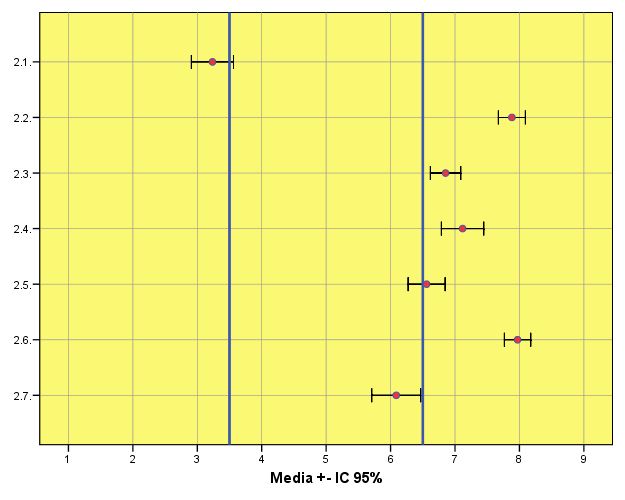


Topic Area 3.- Psychotic depression

|  | **Consensus Disagreement** | **No consensus** | **Consensus Agreement** |
| --- | --- | --- | --- |
| 3.1. Negativistic/oppositional behaviors in depressed older adults point towards the presence of psychotic depression. |  | ◼ |  |
| 3.2. The onset of psychotic symptoms in depressed older adults involves a higher risk for evolving into dementia. |  |  | ◼ |
| 3.3. The onset of psychotic symptoms in depressed older adults involves a greater risk for suicide. |  |  | ◼ |
| 3.4. ECT is the first therapeutic option to treat psychotic depression in the elderly. |  | ◼ |  |
| 3.5. Combined treatment with antidepressants and antipsychotics is the first therapeutic option to treat psychotic depression in the elderly. |  |  | ◼ |
| 3.6. ECT should ONLY be used to treat psychotic depression in older adults when there is lack of response to pharmacological treatment. | ◼ |  |  |
| 3.7. In psychotic depression in the elderly, dual-action antidepressants are preferable to SSRIs. |  |  | ◼ |
| 3.8. When pharmacological treatment fails, ECT is the best option to treat psychotic depression in the elderly. |  |  | ◼ |
| 3.9. In non-life-threatening psychotic depression in the elderly that does not respond to pharmacological treatment, ECT should be prescribed no later than 6 weeks. |  | ◼ |  |
| 3.10. In non-life-threatening psychotic depression in the elderly that does not respond to pharmacological treatment, ECT should be prescribed no later than 12 weeks. |  | ◼ |  |
| 3.11. In non-life-threatening psychotic depression in the elderly that does not respond to pharmacological treatment, ECT should be prescribed no later than 24 weeks. | ◼ |  |  |
| 3.12. After good response to ECT in the acute phase, pharmacological treatment combined with continuation /maintenance ECT is the treatment of choice to prevent early relapse and recurrence. |  |  | ◼ |
| 3.13. After good response to ECT in the acute phase, pharmacological-only continuation /maintenance treatment is the treatment of choice to prevent early relapse and recurrence. |  | ◼ |  |
| 3.14. After good response to ECT in the acute phase, continuation /maintenance ECT therapy-only is the treatment of choice to prevent early relapse and recurrence. |  | ◼ |  |
| 3.15. After good response to ECT in the acute phase, the addition of lithium to the combined antidepressant-antipsychotic drug therapy is the treatment of choice to prevent early relapse and recurrence. | ◼ |  |  |
| 3.16. During continuation /maintenance treatment of psychotic depression, the antipsychotic should be maintained as long as the antidepressant. | ◼ |  |  |
| 3.17. In psychotic depression in the elderly, pharmacological treatment should be maintained indefinitely even if there has only been one episode. |  | ◼ |  |

Topic Area 3.- Psychotic depression. Degree of agreement analysis


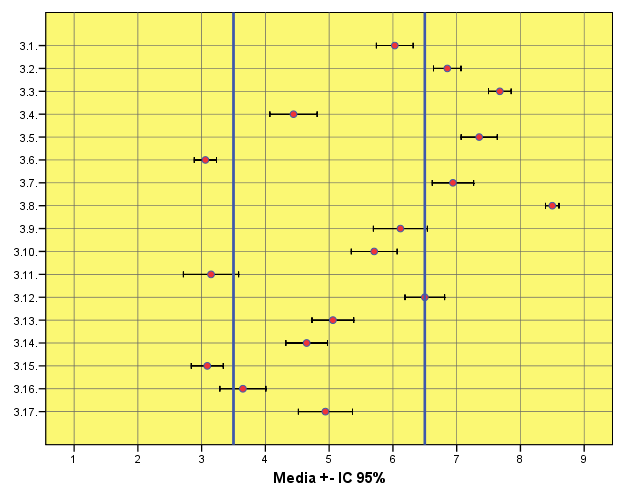


Topic Area 4.- Depression and dementia

|  | **Consensus Disagreement** | **No consensus** | **Consensus Agreement** |
| --- | --- | --- | --- |
| 4.1. The criteria to diagnose depression in dementia/major neurocognitive disorder are well defined and clinically useful. | ◼ |  |  |
| 4.2. It is necessary to establish differentiated depression criteria for the different diseases or clinical conditions that may involve dementia/major neurocognitive disorder (for example, Alzheimer’s disease, Parkinson’s disease, frontotemporal dementia, etc.). |  |  | ◼ |
| 4.3. It is necessary to establish differentiated depression criteria for the different phases of dementia/major neurocognitive disorder. |  |  | ◼ |
| 4.4. Antidepressant drugs are effective in the treatment of depression in dementia. |  |  | ◼ |
| 4.5. Psychological therapies are effective in the treatment of depression in dementia. |  |  | ◼ |

Topic Area 4.- Depression and dementia. Degree of agreement analysis


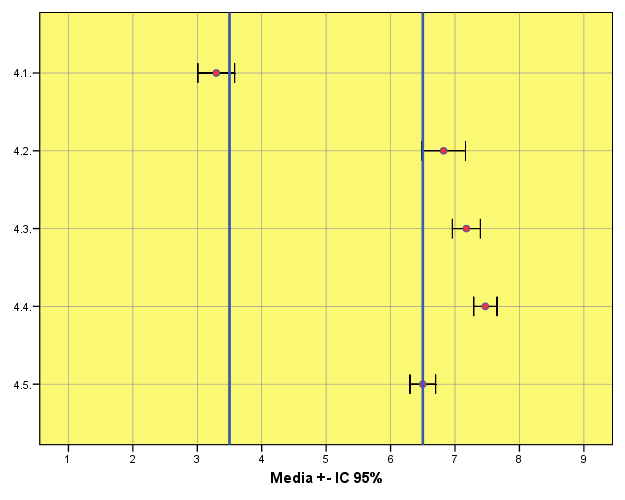


Topic Area 5.- Antidepressant drug treatment

|  | **Consensus Disagreement** | **No consensus** | **Consensus Agreement** |
| --- | --- | --- | --- |
| 5.1. Subclinical depression requires pharmacological treatment. |  | ◼ |  |
| 5.2. When prescribing antidepressants for elderly patients, laboratory tests should be carried out at the beginning of treatment. |  |  | ◼ |
| 5.3. When prescribing an antidepressant for an elderly patient, an EKG should be performed at the beginning of treatment. |  |  | ◼ |
| 5.4. In general, SSRIs antidepressants are first choice treatment drugs for depression in the elderly. |  |  | ◼ |
| 5.5. In general, dual-action antidepressants are first choice drugs for depression in the elderly. |  | ◼ |  |
| 5.6. Dual-action antidepressants achieve higher levels of effectiveness in the treatment of depression in the elderly as compared to SSRIs. |  |  | ◼ |
| 5.7. Dietary supplements (Omega 3, DHA...) are effective in improving depression in the elderly. |  | ◼ |  |
| 5.8. Antidepressants have a slower onset of action in the elderly than in younger adults. |  |  | ◼ |
| 5.9. The introduction of antidepressants increases the risk for suicide in depressed elderly patients at the beginning of treatment. |  | ◼ |  |
| 5.10. The elderly tolerate dual-action antidepressants better than SSRIs. |  | ◼ |  |
| 5.11. Sexual dysfunction caused by antidepressants is a problem for elderly patients. |  |  | ◼ |
| 5.12. After a depressive episode in an elderly patient, treatment at effective doses should be maintained for 6 months. |  | ◼ |  |
| 5.13. After a depressive episode in an older adult, treatment at effective doses should be maintained for 1 year. |  |  | ◼ |
| 5.14. After a depressive episode in an older adult, treatment at effective doses should be maintained for 2 years. |  | ◼ |  |
| 5.15. After a depressive episode in an older adult, treatment at effective doses should be maintained for 3 to 4 years. | ◼ |  |  |
| 5.16. In depression in elderly patients, indefinite pharmacological therapy should be stablished after the first episode. | ◼ |  |  |
| 5.17. In depression in elderly patients, indefinite pharmacological therapy should be stablished after the second episode. |  |  | ◼ |
| 5.18. In depression in elderly patients, indefinite pharmacological therapy should be stablished after the third episode. |  |  | ◼ |

Topic Area 5.- Antidepressant drug treatment. Degree of agreement analysis


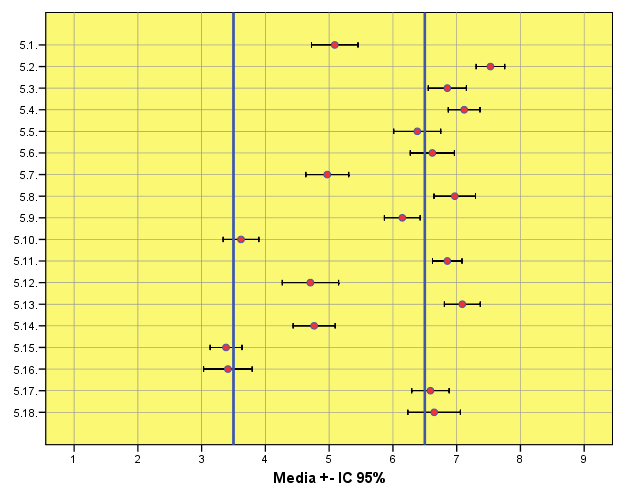


Table 7 Non-pharmacological biological treatments (fundamentally ECT)

|  | **Consensus Disagreement** | **No consensus** | **Consensus Agreement** |
| --- | --- | --- | --- |
| 6.1. ECT is indicated for vascular depression. |  |  | ◼ |
| 6.2. ECT is indicated for depression in elderly patients with dementia. |  |  | ◼ |
| 6.3. Bilateral ECT should only be used when unilateral ECT yields unsatisfactory results. | ◼ |  |  |
| 6.4. Cognitive effects associated with ECT significantly limit its indication for depression in elderly patients. | ◼ |  |  |
| 6.5. Transcranial magnetic stimulation should be regarded as a therapeutic option for resistant depression in elderly patients. |  | ◼ |  |

Topic Area 6.- Non-pharmacological biological treatments (fundamentally ECT). Degree of agreement analysis


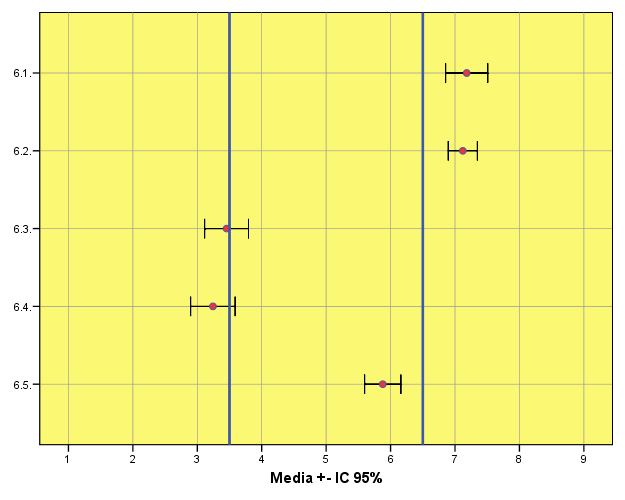


Topic Area 7.- Psychotherapeutic treatments

|  | **Consensus Disagreement** | **No consensus** | **Consensus Agreement** |
| --- | --- | --- | --- |
| 7.1. The efficacy of psychotherapy in geriatric depression is at least equal to that of pharmacological treatments. |  | ◼ |  |
| 7.2. Psychotherapy is less effective in the elderly than in adults. |  | ◼ |  |
| 7.3. In geriatric depression, the presence of cognitive impairment/mild dementia does not limit the use of psychotherapy. |  |  | ◼ |
| 7.4. Psychotherapy is effective in the treatment of subclinical geriatric depression. |  |  | ◼ |

Topic Area 7.- Psychotherapeutic treatments. Degree of agreement analysis


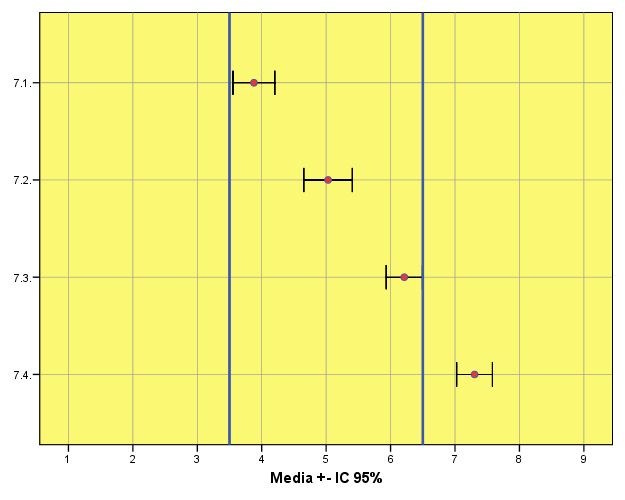


Topic Area 8.- Comorbidity and preventive aspects

|  | **Consensus Disagreement** | **No consensus** | **Consensus Agreement** |
| --- | --- | --- | --- |
| 8.1. In cases of late-onset depression it is necessary to consider the subsequent development of a neurodegenerative disorder (dementia, Parkinson’s disease, etc.). |  |  | ◼ |
| 8.2. In cases of late-onset depression it is necessary to consider the subsequent development of a major medical condition (cancer, cardiopathies, etc.). |  |  | ◼ |
| 8.3. Elderly patients living in nursing homes have access to the same therapies (antidepressant treatment, psychotherapies, ECT, etc.) as those living in the community. | ◼ |  |  |
| 8.4. Teleassistance services are useful to reduce the risk for suicide in the elderly. |  |  | ◼ |
| 8.5. Physical exercise has a significant protective effect against depression in old age. |  |  | ◼ |
| 8.6. Interventions aimed at reducing social isolation are significant and effective strategies in the prevention of depression in the elderly. |  |  | ◼ |

Topic Area 8.- Comorbidity and preventive aspects. Degree of agreement analysis


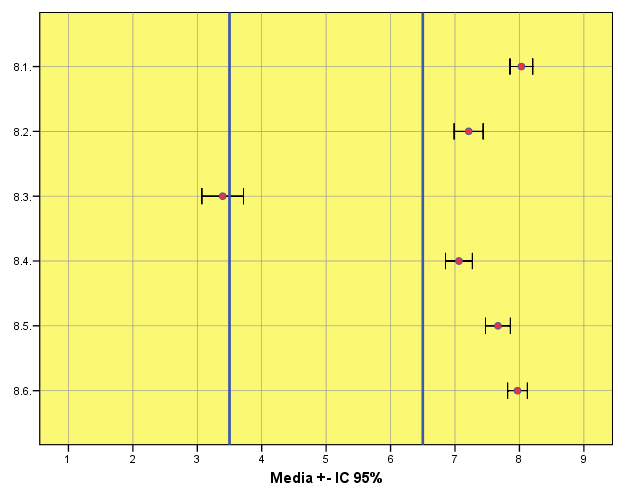


Topic Area 9.- Professional training to address depression in the elderly

|  | **Consensus Disagreement** | **No consensus** | **Consensus Agreement** |
| --- | --- | --- | --- |
| 9.1. The training received by residents in psychiatry is currently insufficient to competently address depressive disorders in older adults. | ◼ |  |  |
| 9.2. Psychiatrists require specific training to competently address depressive disorders in older adults. |  |  | ◼ |
| 9.3. Detection of depression in older adults is currently noticeably below optimal and desirable levels. |  |  | ◼ |
| 9.4. Because of its complexity, depression on older adults should be mainly treated by specialists in psychiatry. |  |  | ◼ |

Topic Area 9.- Professional training to address depression in the elderly. Degree of agreement analysis


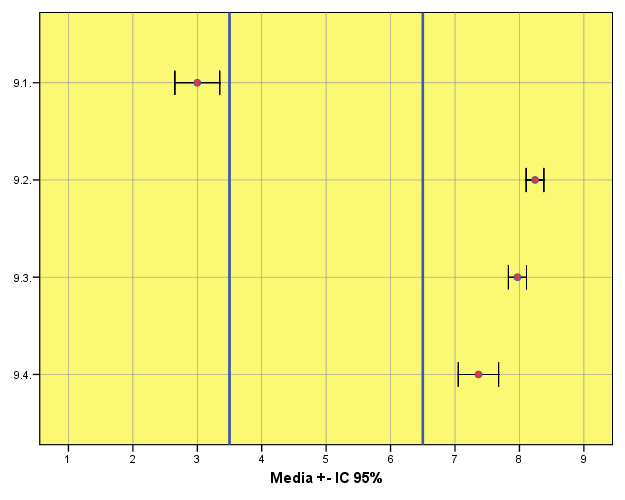

Supplement: Supplementary file 1 [file Table_1.docx]
